# *Pparγ1* Facilitates ErbB2-Mammary Adenocarcinoma in Mice

**DOI:** 10.3390/cancers13092171

**Published:** 2021-04-30

**Authors:** Xuanmao Jiao, Lifeng Tian, Zhao Zhang, Joanna Balcerek, Andrew V. Kossenkov, Mathew C. Casimiro, Chenguang Wang, Yichuan Liu, Adam Ertel, Raymond E. Soccio, Eric R. Chen, Qin Liu, Anthony W. Ashton, Wei Tong, Richard G. Pestell

**Affiliations:** 1 Pennsylvania Cancer and Regenerative Medicine Research Center, Baruch S. Blumberg Institute, Wynnewood, PA 19096, USA; xuanmao.jiao@bblumberg.org (X.J.); gdzhangzhao@163.com (Z.Z.); Mathew.casimiro@abac.edu (M.C.C.); ashtona@mlhs.org (A.W.A.); 2 Department of Cancer Biology, Thomas Jefferson University, Philadelphia, PA 19107, USA; tianlifeng1976@gmail.com (L.T.); chenguangwang@yahoo.com (C.W.); adam.ertel@jefferson.edu (A.E.); 3 Division of Hematology, Children’s Hospital of Philadelphia, Philadelphia, PA 19104-4318, USA; joannabk85@gmail.com (J.B.); tongw@email.chop.edu (W.T.); 4 Center for Systems and Computational Biology, The Wistar Institute, Philadelphia, PA 19107, USA; akossenkov@wistar.org (A.V.K.); qliu@wistar.org (Q.L.); 5 Department of Science and Mathematics, Abraham Baldwin Agricultural College, Tifton, GA 31794, USA; 6 Center for Applied Genomics, Children’s Hospital of Philadelphia, Philadelphia, PA 19146, USA; axl1999@gmail.com; 7 Division of Endocrinology, Diabetes, and Metabolism, Department of Medicine, Department of Genetics, and the Institute for Diabetes, Obesity, and Metabolism, Perelman School of Medicine at the University of Pennsylvania, Philadelphia, PA 19104, USA; soccio@mail.med.upenn.edu (R.E.S.); erc80@case.edu (E.R.C.); 8 Lankenau Institute for Medical Research, Wynnewood, PA 19069, USA

**Keywords:** peroxisome proliferator-activated receptor gamma (PPARγ), nuclear receptor, breast cancer, lipogenesis

## Abstract

**Simple Summary:**

HER2, which is associated with clinically aggressive disease, is overexpressed in 15–20% of breast cancers (BC). Peroxisome proliferator-activated receptor γ (PPARγ), is expressed in a variety of malignancies. The aim of our study was to determine the function of endogenous *Ppar*γ*1* in the onset and progression of mammary tumors induced by ErbB2 in mice. Genetic deletion of *Ppar*γ*1* slowed the rate of tumor progression and death from ErbB2-induced mammary tumors. The deletion of *Ppar*γ*1* correlated with reduced pro-tumorigenic inflammation. We conclude ErbB2 collaborates with endogenous *Pparγ1* in the onset and progression of mammary tumorigenesis.

**Abstract:**

HER2, which is associated with clinically aggressive disease, is overexpressed in 15–20% of breast cancers (BC). The host immune system participates in the therapeutic response of HER2^+^ breast cancer. Identifying genetic programs that participate in ErbB2-induced tumors may provide the rational basis for co-extinction therapeutic approaches. Peroxisome proliferator-activated receptor γ (PPARγ), which is expressed in a variety of malignancies, governs biological functions through transcriptional programs. Herein, genetic deletion of endogenous *Pparγ1* restrained mammary tumor progression, lipogenesis, and induced local mammary tumor macrophage infiltration, without affecting other tissue hematopoietic stem cell pools. Endogenous *Pparγ1* induced expression of both an EphA2-Amphiregulin and an inflammatory INFγ and Cxcl5 signaling module, that was recapitulated in human breast cancer. *Pparγ1* bound directly to growth promoting and proinflammatory target genes in the context of chromatin. We conclude *Pparγ1* promotes ErbB2-induced tumor growth and inflammation and represents a relevant target for therapeutic coextinction. Herein, endogenous *Pparγ1* promoted ErbB2-mediated mammary tumor onset and progression. PPARγ1 increased expression of an EGF-EphA2 receptor tyrosine kinase module and a cytokine/chemokine 1 transcriptional module. The induction of a pro-tumorigenic inflammatory state by *Pparγ1* may provide the rationale for complementary coextinction programs in ErbB2 tumors.

## 1. Introduction

ErbB2 is overexpressed in approximately 25% of human breast cancers, associated with clinically aggressive disease [1]. No soluble ligand has been identified and the receptor is regulated by heterodimerization with other ErbB family receptors, including EGFR, and other receptor tyrosine kinases including EphA2 [2]. EGFR is activated by seven different growth factors including EGF and Amphiregulin [3]. Downstream signaling modules required for ErbB2 induced tumorigenesis in genetically engineered mouse models (GEMM) include the phosphatidylinositol 3-kinase/Akt (PKB) pathway [4], the Ras/Raf/MEK/ERK1/2 pathway and the phospholipase C (PLCγ) pathways [5]. ErbB2-mediated tumorigenesis involves [2,6] activation of receptor tyrosine kinases [5,7], induction of cyclin D1/CDK activity [8], and functional restraint by tumor suppressors [9,10,11]. The receptor tyrosine kinase EPH receptor A2 (EphA2), a member of the Eph RTK family, is overexpressed in aggressive breast cancer and EphA2 forms a complex with ErbB2 thereby enhancing ErbB2-induced tumor onset and progression [7].

HER2-targeted breast cancer treatments, include monoclonal antibodies (trastuzumab, pertuzumab), tyrosine kinase inhibitors (lapatinib, neratinib), and antibody–drug conjugates (Ado-trastuzumab emtansine [T-DM1]) [12,13,14,15]. Herceptin and the dual tyrosine kinase inhibitor Lapatanib, have resulted in dramatic improvements in survival [16], conveyed through both direct and indirect effects which involve anti-tumor immunity [17,18,19]. HER2^+^ breast cancers have higher stromal tumor-infiltrating lymphocytes (TILs) levels than hormone receptor positive (HR^+^)/HER2^-^ breast cancers, correlating with better prognosis [18,20]. HER2-enriched breast cancers are more immunogenic than others (Luminal A/B [21]. As nearly all patients with metastatic Her2-positive breast cancer will progress on treatment it is essential to develop coextinction approaches targeting multiple pathways. The identification of endogenous target genes governing tumor metabolism and inflammation is essential in order to provide a rational approach to extinguishing multiple pathways activated in cancer [22].

The activation of oncogenic signals, downregulation of tumor suppressor pathways, metabolic changes and alterations in the tumor microenvironment, including immune cells, contribute to tumor progression [22,23,24]. The tumor microenvironment (TME) is regulated by chemokines and their G protein coupled receptors binds several ligands, including Cxcl5 which binds Cxcr2, to augment the pro-tumor immune response [25,26], tumor growth and metastasis [27,28]. In this regard, Cxcr2 plays an important role in governing the pro-inflammatory response in mammary tumors inducing the proportion of Gr1^+^ tumor-associated granulocytes, F4/80^+^ tumor-associated macrophages, and CD11b^+^ Gr1^+^ myeloid derived suppressor cells (MDSC) [26]. Tumor-associated macrophages (TAM), which promote tumorigenesis [29], arise primarily from the Ly6C^+^ population of circulating mouse monocytes from the bone marrow [30,31], with a smaller proportion from the spleen [32]. Clinical studies and experimental mouse models indicate that F4/80^+^ macrophages play a pro-tumoral role [33,34,35]. 

Recent studies have suggested an association between Peroxisome proliferator-activated receptor γ (PPARγ) signaling and lapatinib-resistant breast cancer. PPARγ is a member of the nuclear receptor (NR) superfamily that regulates diverse biological functions including lipogenesis and differentiation, inflammation, insulin sensitivity, cellular proliferation and autophagy [36,37,38,39]. Evidence for PPARγ as a tumor growth inhibitor includes the detection of heterozygous *PPARγ* mutations in colon cancer and the finding that PPARγ agonists reduce tumorigenesis in murine models [40,41,42]. In contrast several lines of evidence suggest PPARγ augments growth as PPARγ ligands increased gastrointestinal polyp number in the *Apc* mouse model of familial adenomatosis [43]. Although the preponderance of studies demonstrated PPARγ restrains inflammation [44,45], the impact on anti-tumor immune responses and the role for endogenous *Pparγ* in promoting local tissue and disease-specific inflammatory processes appears to be more complex as a pro-inflammatory effect of *Pparγ* [46], the induction of tissue specific pools of macrophages [47] have been described. Furthermore, studies that deployed ER-Hoxb8-immortalized bone marrow-derived macrophages from *Ppar*γ*^-^*and *LysM-Cre Ppar* *^fl/f^*mice, showed loss of *Pparγ* resulted in reduced F4/80^+^ macrophages [46]. Although *Pparγ* has been implicated in the regulation of cellular growth, the role of endogenous *Pparγ1* in the growth of mammary tumors and the impact on the anti-tumor immune response was not previously known. In order to determine the role of endogenous *Pparγ1* in mammary tumorigenesis the *Pparγ1* gene was deleted in mammary targeted ErbB2 transgenic mice. 

## 2. Results

### 2.1. Deletion of Endogenous PPARγ1 Reduces ErbB2-Induced Mammary Tumor Growth

**Figure 1:**
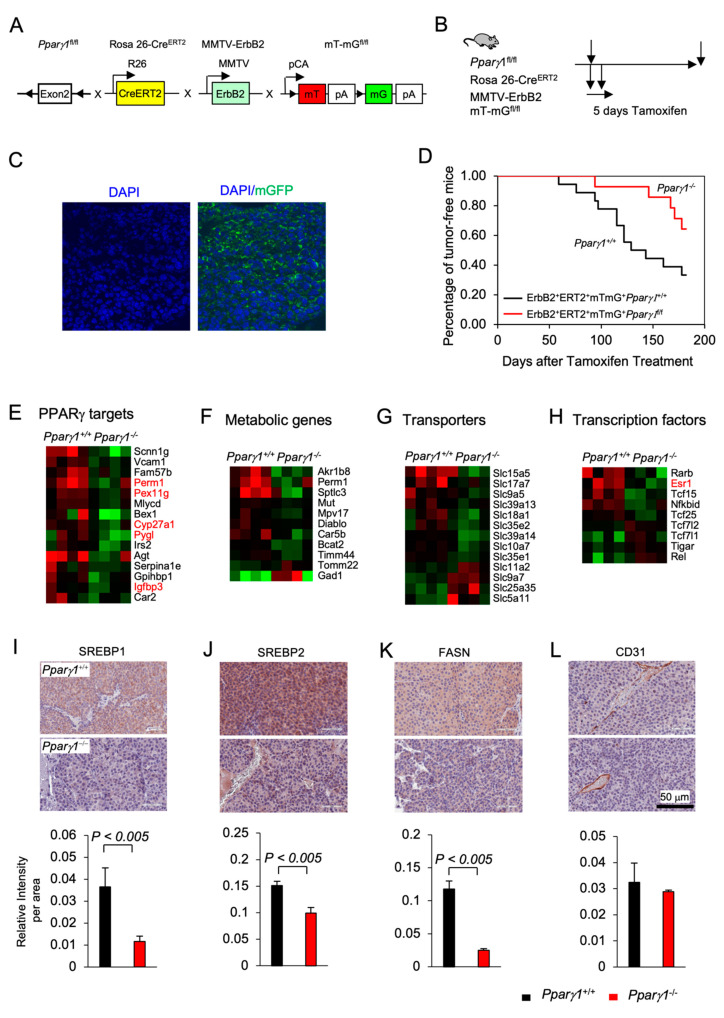
*Pparγ1* collaborates in ErbB2-induced mammary adenocarcinoma, promoting *Pparγ1* signaling. (**A**). Schematic representation of the multigenic mice treatment used to induce Cre excision from the ROSA26 locus using (**B**). 5 days of tamoxifen treatment. (**C**). Representative example of mammary gland tumors showing transgenic fluorescent of GFP reflecting Cre excision. (**D**). Kaplan-Meier curves displaying the onset of tumorigenesis. Data are shown as percentage of tumor free mice. Comparison is made of the *Pparγ1^fl/fl^CreERT2^+^mTmG^+^MMTV-ErbB2* (*Pparγ1^−/−^)* and the *Pparγ1^wt/wt^CreERT2^+^mTmG^+^MMTV-ErbB2* (*Pparγ1^+/+^)* transgenic mice treated with Tamoxifen for 5 days. (**E**). Statistical analysis of the difference in tumor onset rates between the transgenic mice. (**F–I**). Gene expression derived by RNA-seq from tumors of ErbB2 mammary OncoMice with inducible *Pparγ1* deletion showing mean-fold change in levels of expression. Data is shown by functional categories for individual mice tumors as color display with red for increased levels of expression and blue for reduced levels of expression in *Pparγ1*^+/+^ vs. *Pparγ1*^−/−^ mammary tumors. (**J–L**). Representative staining of tumors derived from the transgenic mice with quantitation for SREBP1, SREBP2, FASN, and CD31 with quantitation shown as mean ± SEM for *n* = 3 separate mice for each genotype.

Previous studies demonstrated that PPARγ may either enhance or inhibit tumor growth depending upon the tumor type and oncogenic driver [40,41,42,43]. In order to define the role of endogenous *Pparγ1* in the onset and growth of ErbB2-induced mammary tumorigenesis in an immune-competent context in vivo, we generated multigenic mice in which the *Pparγ1* exon 2 alleles were flanked by LoxP sites and the induction of Cre recombinase was governed through the addition of Tamoxifen (TMX) (Rosa26^ERT2Cre^) with the mT/mG transgenic reporter (The mT/mG transgenic reporter mouse cells that have not undergone recombination express membrane-targeted tdTomato (mT), but after recombination they express membrane-targeted enhanced green fluorescent protein (EGFP)). These mice were intercrossed with the transgenic mammary gland oncomouse MMTV-ErbB2 (Figure 1A) in order to generate transgenic mice, *Pparγ1^fl/fl^CreERT2^+^mTmG^+^MMTV-ErbB2^+ (^Pparγ1^−/−^),* and the control mice, *Pparγ1^wt/wt^CreERT2^+^mTmG^+^MMTV-ErbB2^+^(Pparγ1^+/+^).* The multigenic offspring were treated with a 5 days pulse of Tamoxifen (Figure 1B and S2A), which was sufficient to induce Cre expression, deletion of the target gene and conversion of the tdTomato expressing mammary epithelium to EGFP (Figure 1C). Tumor formation was compared between the transgenic mice *(Pparγ1^−/−^* vs. *Pparγ1^+/+^*) treated with Tamoxifen with analysis conducted over the subsequent 6 months (Figure 1D). Statistical analysis demonstrated that the rate of MMTV-ErbB2 induced mammary tumor onset was delayed in mice in which the *Pparγ1* gene had been deleted through Cre recombinase (by the log-rank test, *p* = 0.043; by Wilcoxon rank-sum test, *p* = 0.028 or by the Tarone-Ware test, *p* = 0.033) (Table S1). The potential independent effect of Tamoxifen on mammary tumor rate was controlled for, as each line received identical exposure to tamoxifen. Growth rate of established ErbB2 mammary tumors upon Cre-mediated deletion of *Pparγ1* was determined as well. Tumor volumes in MMTV-ErbB2, *ROSA26^CreERT2/mTmG^* mice that were either *Pparγ1^+/+^* or *Pparγ1^−/−^* were showed in Figure S2B (*n* = 4 of each genotype). The estimated tumor growth rate for each group was calculated based on the mixed effects models and showed in Table S2. The results showed that tumor growth in *Pparγ1^−/−^* mice was slower than in *Pparγ1^+/+^* mice.

In order to determine the molecular mechanisms by which *Pparγ1* maintained ErbB2 mammary tumors, analysis of RNA-seq based gene expression derived from the *Pparγ1^−/−^* vs. *Pparγ1^+/+^* mammary tumors was conducted, revealing deletion of endogenous *Pparγ1* changed substantially (up to 24-fold) the levels of gene expression (Figure S1A), with reduced expression of *Pparγ1-*responsive genes (*PERM1, (Peroxisome Proliferator-Activated Receptor Gamma Coactivator 1 And Estrogen-Related Receptor-Induced Regulator In Muscle 1), PEX11G* (Peroxisomal Biogenesis Factor 11 Gamma), *Cyp27A1* and *Pygl1 (glycogen phosphorylase1* (Figure 1E), and reduced lipogenic and metabolic genes (Figure 1F). Consistent with reduced anabolism and lipogenesis *Pparγ1^−/−^ ErbB2* tumors showed reduced expression of anabolic solute carriers (Figure 1G) and reduced expression of growth promoting transcription factors including reduced nuclear receptors *Esr1* (*ER*α), *Tcf25*, and *Tcf15* (Figure 1H). The abundance of key enzymes required for *de novo* lipogenesis, including sterol regulatory element-binding protein (SREBP1, SREBP2) and fatty acid synthase (FASN) was reduced in *Pparγ1^−/−^* vs. *Pparγ1^+/+^* ErbB2 mammary tumors (Figure 1I–K and Figure S1B). CD31 (cluster of differentiation 31) expression was not significantly changed in endothelial cells (Figure 1L).

The MCF10A-NeuT-PPARγ1 cells showed reduced transwell migration compared with vector control. In order to determine the role of PPARγ1 in ErbB2 induced growth in xenografts, MCF10A-NeuT cells were transduced with PPARγ1 or vector control as indicated in Figure S2D. 5 × 10^6^ cells were implanted into the mammary fat pad of immune-deficient nude mice. Tumor growth, measured at 20 days by digital caliper, showed a substantial increase in tumor volume (Figure S2E, Data are shown as mean ± SEM for *n* = 13).

### 2.2. Pparγ1 Augments an EphA2-amphiregulin Growth Gactor Signaling Module

**Figure 2:**
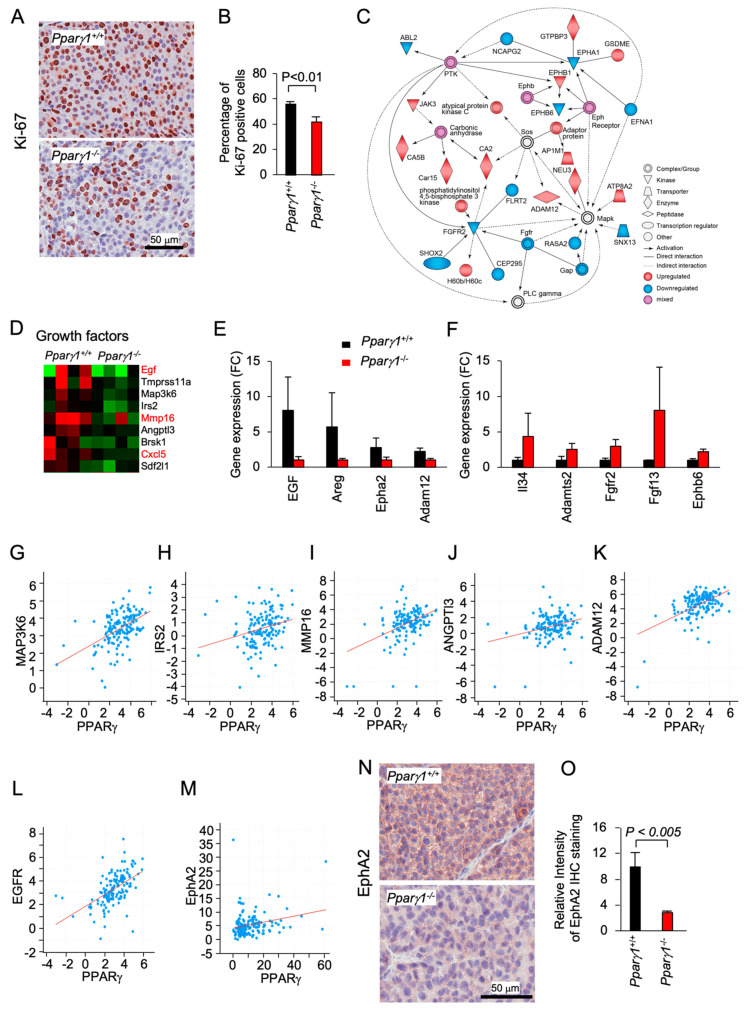
Pparγ induction of EphA2-Areg growth factor signaling in ErbB2-induced mammary adenocarcinoma. The ErbB2 adenocarcinoma were analyzed for (**A**). Ki-67 by immunohistochemical staining with (**B**) quantitation as mean + SEM from *n* = 5 separate mice (*p* < 0.01). (**C**). IPA pathway analysis identified a gene interaction network with upregulation (red) of signaling nodes for PTK (protein tyrosine kinase), SOS and Eph receptor, with down-regulation (blue) for Fgfr2 and Fgfr. (**D**). RNA-seq expression for genes within the “growth factors” KEGG category shown as a heat map display or (**E,F**). as mean ± SEM for *n* = 8 mice. (**G–M**). Correlative gene expression analysis in breast cancer samples (The Metastatic Breast Cancer Project (Provisional, February 2020) between Pparγ and growth factors identified in (**D**). Data are shown for correlation between Pparγ and MAPK3K6 (*n* = 136, Pearson 0.50, *p* = 1.96 × 10^−10^), IRS2 (*n* = 136, Pearson 0.26, *p* = 1.27 × 10^−3^), MMP16 (*n* = 136, Pearson 0.42, *p* = 9.77 × 10^−8^), ANGPT13 (*n* = 136, Pearson 0.20, *p* = 3.3 × 10^−4^), ADAM12 (*n* = 136, Pearson 0.52, *p* = 1.62 × 10^−11^), EGFR (*n* = 136, Pearson 0.53, *p* = 1 × 10^−11^) and EphA2 (*n* = 136, *p* = 3.1 × 10^−3^, Pearson, 0.24). (**N,O**). Representative Immunohistochemical staining for EphA2 shown as (**O**). mean ± SEM for *n* = 8 mice.

Ki-67 staining, a marker of cellular proliferation, demonstrated an increased percentage of Ki-67^+^ staining cells in the *Pparγ1^+/+^* ErbB2 mammary adenocarcinoma (Figure 2A,B). Pathway analysis showed *Pparγ*1 augmented the activity of an Eph receptor node and a protein tyrosine kinase node, which in turn activated atypical PKC, Neu3 and ADAM12, whereas FGFR2 signaling was restrained (Figure 2C). RNA-seq identified the induction of a growth factor module regulators including *EGF, Amphiregulin, IRS2, AngP2, CXCl5, and SDF2l1* (Figure 2D). The abundance of mRNA encoding growth factors known to enhance ErbB2 signaling, including Egf, Amphiregulin, EphA2, and Adam12 was increased (Figure 2D). ADAM12 (a disintegrin and metalloproteinase 12), is linked to the induction of EphA2-dependent cell migration [48]. EphA2, which is known to augment ErbB2-induced mammary tumorigenesis [7], was increased 4-fold in the *Pparγ1*^+/+^ ErbB2 mammary tumors (Figure 2E). EphB6 signaling, known to suppress breast cancer cell aggressiveness by interacting with EPHB4 and interfering with EPHB4 action [49], was repressed by *Pparγ1* in ErbB2 mammary tumors (Figure 2F). In order to examine further the relationship between Ppar*γ* and the abundance of the growth factors maintained by endogenous *Pparγ1* in the murine mammary tumors, we interrogated a public database (https://www.cbioportal.org/study/summary?id=brca_mbcproject_wagle_2017 (accessed on 2–15 December 2020)) in which mRNA expression of breast cancer samples had been determined. These analysis revealed a significant correlation in human metastatic breast cancer between *Pparγ1* and the key growth factors identified (Figure 2G–M), including MAPK3K6 (*n* = 136, Pearson 0.50, *p* = 1.96 × 10^−10^), IRS2 (*n* = 136, Pearson 0.26, *p* = 1.27 × 10^−3^), MMP16 (*n* = 136, Pearson 0.42, *p* = 9.77 × 10^−8^), ANGPT13 (*n* = 136, Pearson 0.20, *p* = 3.3 × 10^−4^), ADAM12 (*n* = 136, Pearson 0.52, *p* = 1.62 × 10^−11^), EGFR (*n* = 136, Pearson 0.53, *p* = 1 × 10^−11^) and EphA2 (*n* = 136, *p* = 3.1 × 10^−3^, Pearson, 0.24). EphA2 abundance, assessed by immunohistochemistry, was increased approximately 5-fold (Figure 2N,O). Collectively these studies are consistent with a model in which endogenous Pparγ1 maintains the abundance of a growth promoting module (ADAM12, EGFR, EphA2), each component of which is known to enhance ErbB2 function.

### 2.3. Pparγ1 Governs the Mammary Tumor Immune Response

**Figure 3:**
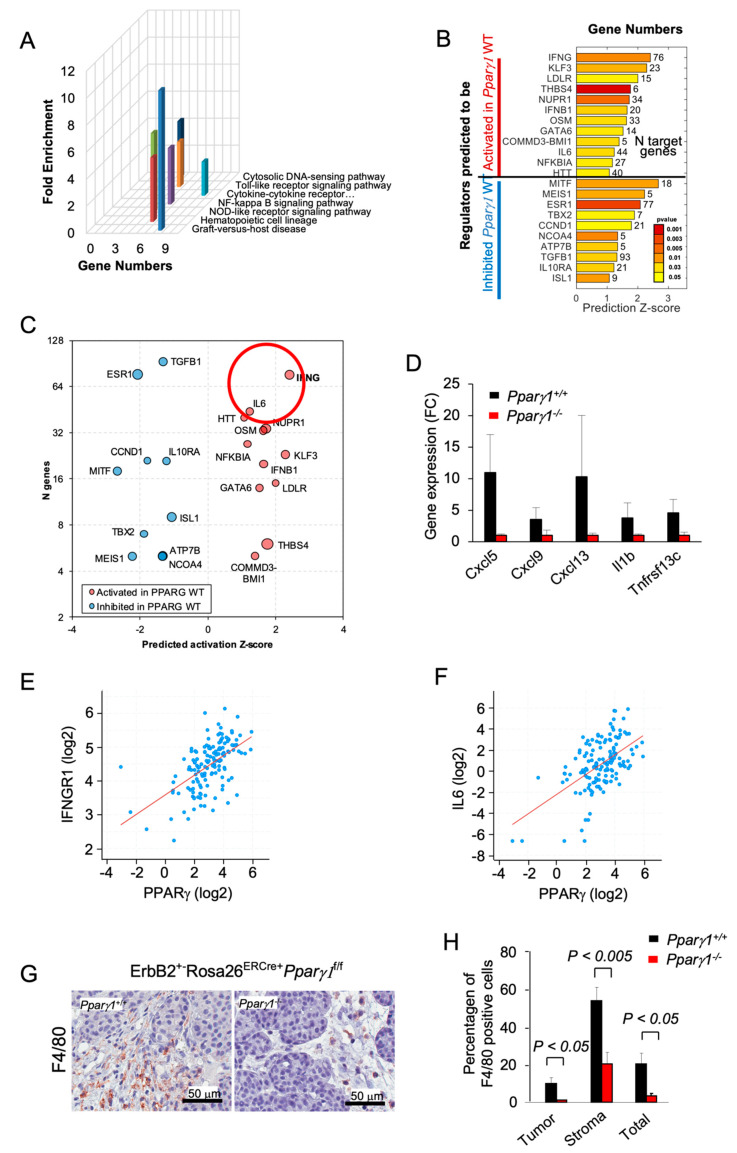
Deletion of *Pparγ1* in MMTV-ErbB2 mammary tumors reduces gene expression of signaling via cytokine/chemokine and growth factor pathways. (**A**). KEGG pathway analysis gene expression from tumors of ErbB2 mammary GEMM with inducible *Pparγ1* deletion showing mean-fold change in levels of expression. (**B,C**). Ingenuity Pathway Analysis (IPA) performed for mammary tumors using “Upstream Regulators” option. The results show regulators with a significant number of changed known targets and *Z*-score for the predicted change of the regulator’s activation state. In (B) *Z*-scores are shown as bars with *p*-values shown in color scale from red (best *p*-value) to yellow. In (**C**) results are represented by bubble plot of *Z*-scores vs. Number of genes (*n*) with size proportional to log_10_(*p*-value) and color indicating predicted increased (red) or decreased (blue) activity of the regulators in *Pparγ1*^+/+^ vs. *Pparγ1*^−/−^ mammary tumors. Lower *p*-values are related to a more significant of number of changed targets of a regulator, and a higher *Z*-score indicates a better evidence of activation or inhibition of regulator’s activity based on the targets direction of change. (**D**). Gene expression for cytokines/chemokines upregulated by Pparγ1 from KEGG analysis. (**E,F**). Correlative gene expression analysis in breast cancer samples from The Metastatic Breast Cancer Project (Provisional, February 2020) between Pparγ and IFNG and IL-6 identified in (**C**). Correlation between *PPARγ* expression and expression of *IFNGR1* (*n* = 136, Pearson 0.57, *p* = 17.13 × 10^−14^), and *IL6* (*n* = 136, Pearson 0.55, *p* = 6.1 × 10^−13^) is highly significant. (**G**). Immunohistochemical staining for the tissue-associated macrophages with F4/80. (**H**). with data shown as mean ± SEM for *n* = 6 separate tumors.

In order to determine the signal transduction pathways maintained by *Pparγ1* in the ErbB2 mammary tumors, we conducted Kyoto Encyclopedia of Genes and Genomes (KEGG) pathway analysis of RNA-seq from the tumors. Deletion of *Pparγ1* reduced activity of the pathways “cytokines/chemokine receptors signaling” and the related “Graft versus Host disease”, “NOD-like receptor signaling” and “NFκB signaling” (Figure 3A). Analysis of significantly affected genes by Ingenuity Pathway Analysis (IPA) identified potentially altered upstream regulators related to cancer and the inflammatory response. INFγ-induced genes were decreased by deletion of endogenous *Pparγ1* (Figure 3B,C), consistent with the known induction of Interferons (IFNs) and IFN-stimulated genes by peroxisomes [50,51]. *Pparγ1*^+/+^ ErbB2 mammary tumors showed increased expression of specific chemokines and cytokines (Cxcl5, Cxcl19, Cxcl13, IL1b and Tnfrsf13c (Figure 3D)). As IFNG and IL6 were the most induced by number of genes and Z score (circled in Figure 3C), we examined the relationship between Pparγ and IFNG and IL6 in mRNA expression data from The Metastatic Breast Cancer Project (Provisional, February 2020) (Figure 3E,F). These analyses revealed a significant correlation in human metastatic breast cancer between Pparγ and IFNGR1 (*n* = 136, Pearson 0.57, *p* = 17.13 × 10^−14^), and with IL6 (*n* = 136, Pearson 0.55, *p* = 6.1 × 10^−13^). The F4/80^+^ marker of murine tissue associated macrophages (TAM) confirmed a significantly increased proportion in the *Pparγ1^+/+^* tumor population within both the tumor and the tumor stroma (Figure 3G,H). Collectively these findings are consistent with a model in which endogenous Pparγ1 maintains an inflammatory tumor microenvironment that includes an increase in F4/80^+^ macrophages.

### 2.4. Pparγ1 in B Cell Differentiation

TAM can be recruited from inflammatory monocytes, tissue generated macrophages [52,53], and can be generated from Pre/proB cells during inflammation [54]. These monocytes are derived from primarily the bone marrow and also the spleen [32,55]. In order to determine whether the changes in the tumor immune environment were a function of Pparγ1 on the hematopoietic system, we conducted a detailed analysis of the hematopoietic cell system. The hematocrit (HCT), counts of white blood cells (WBC), red blood cells and platelets were not significantly changed (Figure S3A–D). The proportion of WBC subtypes in the peripheral blood were unchanged (Figure S3E). The F4/80^+^ macrophage and Mac1^+^Gr1^−^ cellularity counts in the bone marrow, blood and spleen and were unchanged (Figure S3F,G). The spleen showed a modest by significant increase in the percentage of Mac1^+^Gr1^+^ granulocytes (Figure S3H).

**Figure 4:**
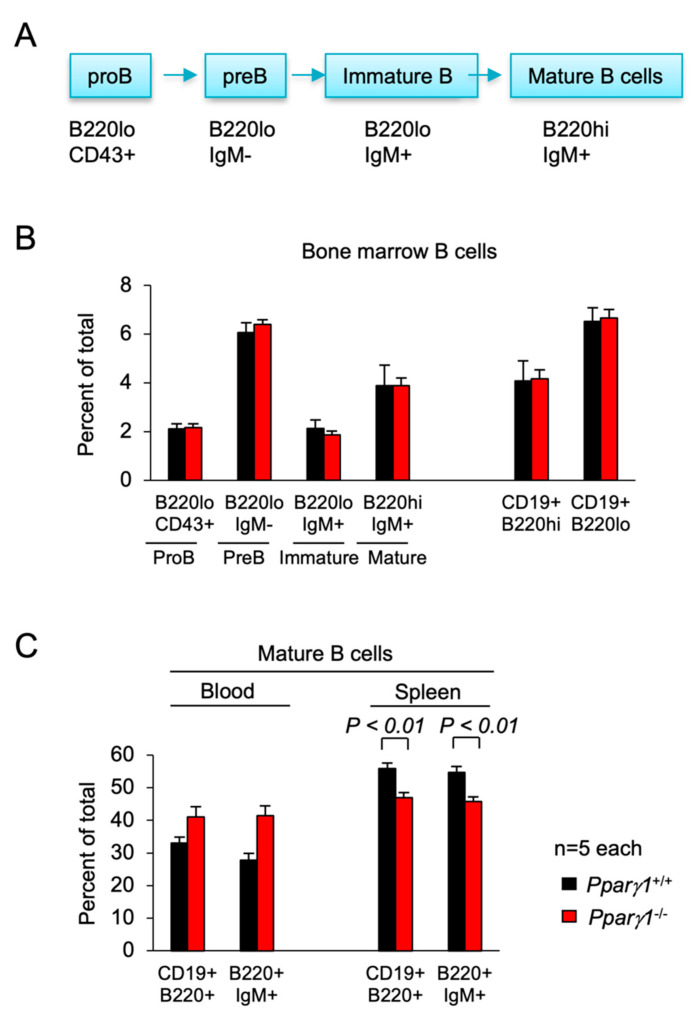
B cell differentiation in *Pparγ1*^+/+^ vs. *Pparγ1*^−/−^ multigenic mice. (**A**). Schematic representation of lineage differentiation from pro B cells to mature B cells. (**B**). Lineage indices of *Pparγ1*^+/+^ vs. *Pparγ1*^−/−^ multigenic mice (*Pparγ1^wt/wt^*, vs. *Pparγ1^fl/fl^*, both in *ROSA26^CreERT2^*, *Rosa26^mTmG^* and *MMTV-ErbB2* background and treated with tamoxifen) with relative distribution of B cell lineage components in the bone marrow (BM). Relative proportion of Pro-B, Pre-B, Immature B cells, and Mature B cells (B220^high^IgM^+^) are shown. (**C**). The proportion of Mature B cells are shown for the blood and spleen. Data are represented as mean ± SEM. *n* = 10 total.

Subsorting for the proportion of ProB, PreB, immature and mature B cells in the bone marrow did not show significant differences between *Pparγ1* genotypes (Figure 4A,B). Analysis of the B-cell lineage in the spleen showed a decrease in the proportion of mature splenic B cells in *Pparγ1^−/−^* mice (B220^hi^CD19^+^) (54.7 vs. 45.7, *p* < 0.05, Figure 4C, *n* = 10 total mice). There was no significant change in the CD4^−^CD8^+^ T cell compartment (Figure S4).

### 2.5. Pparγ1 Does Not Influence Hematopoietic Stem Cell Population (HSC) Differentiation

**Figure 5:**
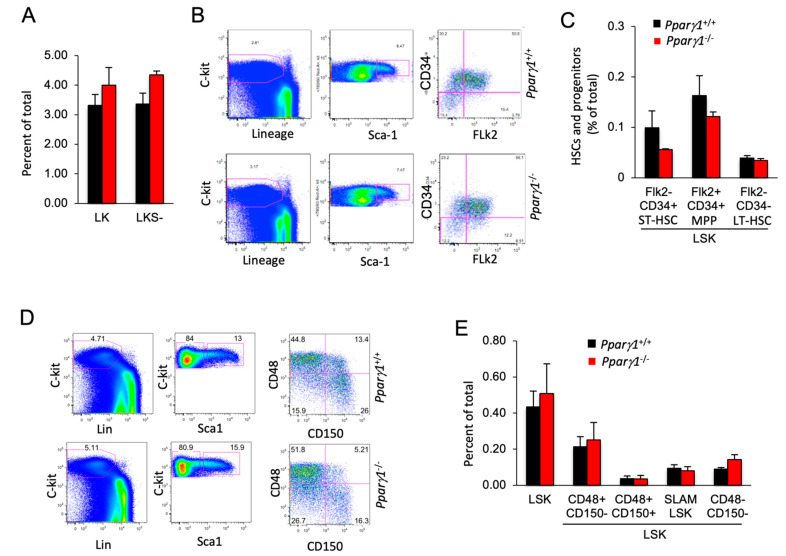
Hematopoietic stem cell (HSC) distribution of LT-HSC and ST-HSC is unchanged in *Pparγ1*^+/+^ vs. *Pparγ1*^−/−^ multigenic mice. (**A**). the LK and LKS^−^ are shown as a percent of total as mean ± SEM. (**B**). Relative distribution of HSC lineage components, using c-Kit, Sca-1 and Flk2 shown as representative FACS analysis. (**C**). HSC lineage proportion of LSK cells shown for proportion of Flk2/CD34 as mean ± SEM. (**D**). Representative FACS plots for the HSC and progenitor markers with antibodies to c-Kit, Lin, Sca-1, CD48/CD150. (**E**). Relative distribution of LT-HSC lineage components (LSK/CD48/CD150) shown as mean ± SEM for *n* = 10 total.

Given the small but significant reduction in the mature B cell population, we assessed the impact of Pparγ1 on the hematopoietic stem cell population (HSC). In the adult mouse, all multipotent cells are contained in the Lineage^−/low^Sca-1^+^c-Kit^+^ (LSK) fraction of bone marrow cells, which was similar between Pparγ1 genotypes (Figure 5A). Assays using flow cytometry to quantify HSPC (Flk2/CD34) demonstrated that HSPCs were unaltered although there was a trend towards a reduction in the Flk2^−^CD34^+^ (ST-HSC or short term HSC) population in *Pparγ1*^−/−^ (Figure 5B,C). The distribution of LSK CD34^−^Flk2^−^ (representing long term-HSC) and LSK CD34^+^Flk2^+^ multipotent progenitors (MPP) (Figure 5B,C) was unchanged.

SLAM family markers, CD150, CD48, CD229, and CD244, can distinguish HSCs and MPPs from restricted progenitors and subdivide them into a hierarchy of functionally distinct subpopulations with stepwise changes in cell-cycle status, self-renewal, and reconstituting potential [56]. The frequency of hematopoietic stem cells and progenitors (HSPCs) assessed by SLAM markers (LSK CD150/CD48) (Figure 5D,E), was unchanged. A quantification of colony forming progenitors and colony subtypes types (M, G, GM, GEMM; (granulocyte (G), erythrocyte, monocyte, megakaryocyte)) [57] demonstrated *Pparγ1^+/+^* did not affect colony forming ability (Figure S5A,B). As Pparγ did not increase the proportion of F4/80^+^ macrophages in the bone marrow, blood or spleen, these studies suggest the reduction in tumor-associated F4/80^+^ macrophages within the ErbB2 *Pparγ1^−/−^* mammary tumors is not due to a Pparγ1-mediated alteration in hematopoietic precursors in the bone marrow or the circulation.

### 2.6. Pparγ1 Binds in Chromatin Immune Precipitation Assays to the Regulatory Regions of the EphA-Amphiregulin and the Chemokine Signaling Axis Genes in Breast Cancer Cells

**Figure 6:**
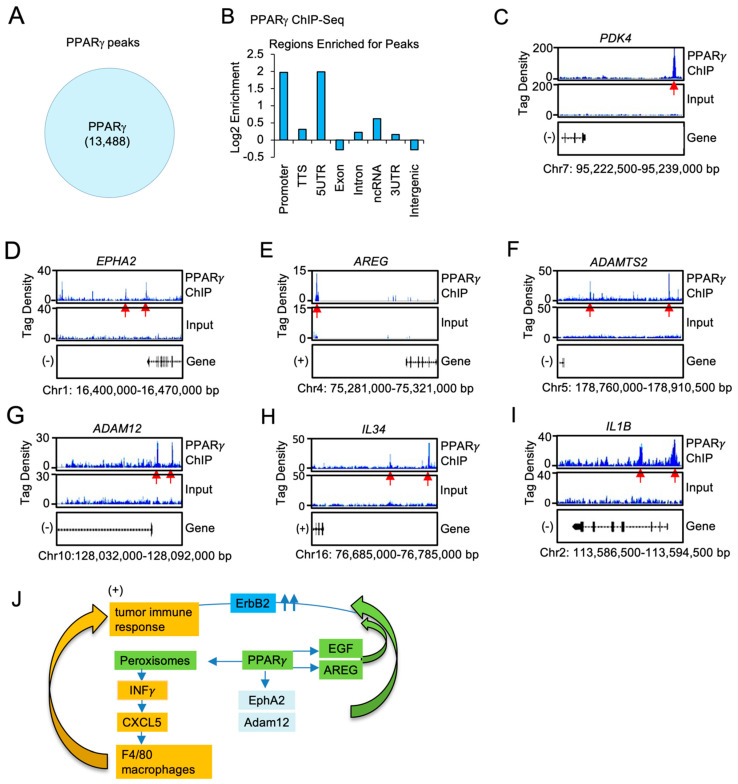
PPARγ*1* binding in chromatin to growth factor and cytokine/chemokine signaling module genes, in ErbB2 mammary tumors. (**A**). ChIP-Seq analysis was performed on samples derived from MCF10A-NeuT transduced with PPARγ Wt and peak calling defined by MACS2 software is shown in the Venn diagram. (**B**). Distribution of PPARγ Wt peaks relative to the TSS. The region upstream from the TSS was divided as indicated. (**C**). Integrated genome browser visualization of tag density profiles for ChIP-Seq PPARγ Wt of selected genes in the PPARγ1 target gene (PDK4), (**D–G**). the growth module *EphA2, Amphiregulin, ADAM12, ADAMTS2* and (**H,I**), *the interferon/cytokine module of IL1B, IL34.* (**J**). Schematic representation of model in which PPARγ1 determines binding to cytokine/chemokine and growth factor genes.

ChIP-Seq was used to define the genome-wide DNA sequence-specific binding characteristics regulated by Pparγ1 in breast cancer cells. High confidence Pparγ1 ChIP-Seq peaks were identified in MCF10A-NeuT cells expressing Pparγ1 Wt. Analysis of the ChIP-Seq data (Figure 6A) demonstrated 13,488 genes were selectively bound by Pparγ1 Wt as defined by the limits of within 10 kb upstream of the transcriptional start site (TSS), 10 kb downstream of the TES and binding within the gene. We next compared the position of ChIP-Seq peaks relative to gene transcription start site and evaluated the number of binding peaks at decreasing intervals upstream from the transcription start site as indicated (Figure 6B). We then determined by ChIP-Seq analysis the binding of PPARγ*1* to the regulatory regions of genes involved in both the growth factor induced module and the inflammatory module. Given the induction by *Ppar*γ*1* of EphA2, Amphiregulin, Adam12 in the mammary tumors we sought to determine whether PPARγ*1* directly interacted in the context of chromatin with these target genes. We first examined the known *Pparγ1* target pyruvate dehydrogenase kinase 4 (PDK4) [58] the expression of which is known to regulate *de novo* lipogenesis. PPARγ1 was enriched in the PDK4 ChIP (Figure 6C). Furthermore PPARγ1 was enriched in the *EPHA2, AREG, ADAMTS, ADAM12* and the *IL34* and *IL1B* ChIP (Figure 6D–I).

## 3. Discussion

The current studies extend our understanding of Her2-function by identifying the critical role for Pparγ1 in mediating ErbB2-mammary tumor progression in vivo. Using tetra-transgenic inducible, gene deletion mice, we show that the *Pparγ1* gene is required for the progression of ErbB2-induced mammary tumorigenesis in immune competent mice. Second, *Pparγ1* gene deletion reduced the expression of genes within signaling pathways mediating growth, including growth factors and growth factor receptors (*EphA2, EGF, Amphiregulin, Adam12*) (Figure 6J). Third, *Pparγ1* gene deletion reduced the influx of an F4/80^+^ macrophage inflammatory infiltrate into the mammary tumors, without affecting hematopoietic cell differentiation. The immune infiltration of the ErbB2 tumors was associated with the induction of lipogenesis, peroxisome signaling, INFγ and Cxcl5 [59]. Collectively these studies suggest Pparγ1 may represent a useful target for coextinction strategies of ErbB2 induced breast cancer.

Previous studies had shown that PPARγ activation by ligands or overexpression may either enhance or inhibit tumor growth depending upon the tumor type and oncogenic driver [40,41,42,43,60,61,62]. Herein, deletion of endogenous Pparγ1 reduced ErbB2-induced mammary tumors. PPARγ antagonists include GW9662 (2-chloro-5-nitro-N-phenylbenzamide) [63], 2-bromo-5-nitro-N-phenylbenzamide [64] and the structurally similar T0070907 [65]. GW9662 and T0070907 have also been reported to produce off-target effects in vitro [66,67,68]. The partial PPARγ agonists include GW0072 [69] and L-764406 [70]. The current studies are consistent with prior studies using GW9662. Mammary carcinogenesis induced by treatment with medroxyprogesterone and dimethylbenz(a)anthracene (DMBA) are ERα-responsive tumors. Continuous administration of GW9662 in the diet enhanced susceptibility to fulvestrant therapy with a marked increase in survival and a reduction in tumor number in the animals maintained on GW9662 and treated with fuvestrant [71].

*Pparγ1* deletion reduced expression of Pparγ1 target genes, genes governing growth factor signaling pathways (*EGF, IRS2, AngP2, CXCl5, SDF2*), transcription factors that promote breast tumor growth including nuclear receptors and anabolic solute carriers (*Slc25a35* (oxaloacetate carrier), *Slc39a13, Slc39a14, Slc15a5, Slc18a1, Slc17a7*) which may participate in cellular growth [72]. The induction of EGF, EphA2 and ADAM12 are known to augment ErbB2-induced tumorigenesis (Figure 6J). EGF is known to enhance ErbB2 signaling via the EGFR/ErbB2 heterodimer [73]. Endogenous Pparγ1 enhanced EphA2 mRNA and protein abundance in the ErbB2 adenocarcinoma. EphA2 is known to enhance ErbB2-induced mammary adenocarcinoma [5,7]. Co-expression of ErbB2 and EphA2 is sufficient to induce tyrosine phosphorylation of EphA2 in the absence of ligand and EphA2 [5,7]. A disintegrin and metalloproteases (ADAMs) are transmembrane metalloproteases that process and shed the ectodomains of membrane-anchored growth factors, cytokines and receptors [74]. ADAM12 is linked to the induction of EphA2-dependent cell migration in metastatic tumors [48]. The membrane type-I matrix metalloproteinase (MT1-MMP) also promotes cancer cell migration and invasion via EphA2 receptor cleavage [75] ADAM12 modulates intracellular signaling by cleaving various membrane bound signaling receptors and their ligands. ADAM12 is highly expressed in glioblastoma multiforme, where it is linked to shedding of HB-EGF. cancer cell invasion, including ADAMs (as reviewed above), and interestingly, ADAM12 [48], and MMP2 and 9 [76] are all upregulated by TGF-β1 signaling in tumor metastasis. ADAM12, ephrin-A1, and EphA2-contribute to growth or cell migration in primary and metastatic tumors [48,77,78].

In order to determine the molecular mechanisms by which Pparγ1 enhanced ErbB2-mediated mammary tumorigenesis tissue culture-based experiments were conducted to examine cell autonomous function. MCF10A-ErbB2-PPARγ1 cells displayed reduced transwell migration compared with vector control. These findings suggest that a cell autonomous effect on migration does not contribute to the tumorigenic phenotype. Prior experiments had supported a model in which Pparγ1 mediated cell non autonomous functions through the induction of secreted factors that promoted angiogenesis [79]. Herein, endogenous Pparγ1 enhanced tumorigenic inflammation. First, Infiltration of F4/80^+^ macrophages into the mammary tumors of transgenic mice, which are known to augment ErbB2-induced tumor progression [80,81], was reduced by *Ppar*γ*1* deletion. EGF, which was increased upon *Pparγ1* gene deletion, is known to augment a feedforward loop within the TME to enhance TAM recruitment [33,34,35]. Interferon signaling (INFγ1, INFβ1), was augmented by endogenous Pparγ1. IPA analysis identified interferon signaling as a predominant pathway induced by Pparγ1. Peroxisomes induced by Pparγ1, activate Type1 interferons (IFNs) and IFN-stimulated gene expression [50,51], including Cxcl5 and Cxcl9. Peroxisome proliferation requires PEX11-type peroxisomal proliferators [82] and herein, endogenous Pparγ1 induced peroxisomal target genes in the mammary tumors as evidenced by increased expression of *PEX-11*, together with *PPARGC1* and *ESRR induced regulator, muscle 1*, *PGC-1 and ERR-induced regulator in muscle 1* (*Perm1*). Cxcl5 is a key local cue that recruits tumor associated macrophages (TAMs), which in turn promote a pro-tumorigenic environment [26,52,53]. CXCL9 which may also be pro-tumorigenic [59], is upregulated during BCa therapy [83] and participates in augmenting the response to cancer therapy with checkpoint blockade [84]. IL-1b [85,86] and IL34, which were induced by Pparγ1 are also known to induce a pro-tumorigenic tumor microenvironment (TME) with IL-34 enhancing recruitment and survival of TAM [87].

In order to determine potential mechanisms by which Pparγ1 coordinated the induction of growth factor and cytokine/chemokine signaling in ErbB2 tumors, we conducted genome wide PPARγ1 chromatin binding assays in human mammary adenocarcinoma cells. The expression of genes governing growth factor (*EphA2, amphiregulin, Adam12, AdamTS2*) and a chemokine module (*CXCL5, IL1B,IL34, TNFRSF13C*), were induced by PPARγ*1* and bound in ChIP to target regions within the genes. PPARγ1 ChIP analysis in MCF10A-NeuT mammary tumors showed the number of sites bound by PPARγ1 Wt including all *cis* elements was comparable to the ~23,000 and ~21,000 sites identified in human adipocyte cell lines [88], but fewer than the ~40,000 sites identified in primary in vitro differentiated human adipose derived stem cells. In adipocytes, C/EBPs co-localizes with PPARγ1 at the majority of its binding sites and cooperate in target gene transcription [88,89], whereas in macrophages, PPARγ1 ChIP is enriched with the hematopoietic transcription factor PU.1 [90]. Collectively these findings are consistent with a model in which Pparγ1 promotes the expression of gene governing lipogenesis, mammary tumor growth and a local tumor immune response. Pparγ1 should be considered further for coextinction paradigms in ErbB2-mediated adenocarcinoma.

## 4. Materials and Methods

### 4.1. Transgenic Mice

Mice were kept on a 12 h light/dark cycle with ad libitum access to chow and water. The *Pparγ1^fl/fl^* mice (which remove exon 2 of the *Pparγ1* locus [91]), ROSA26^CreERT2^ mice (Expressing CRE-ERT2 fusion protein under the control of the ubiquitous *ROSA26* promoter. Cre-ERT2 fusion protein will bind with tamoxifen and then transport to nucleus to induce the deletion of floxed alleles [92]) and ROSA26^mTmG^ mice [93] (ROSA26^mTmG^ is a cell membrane-targeted, two-color fluorescent Cre-reporter allele. Prior to Cre recombination, tdTomato (mT) red fluorescence will be expressed. Followed Cre recombination, instead of the red fluorescence, cell membrane-localized EGFP (mG) fluorescence will be expressed) (Stock No. 007576, The Jackson Laboratory, Bar Harbor, ME, USA) [93] were previously described. Mice homozygous for a floxed *Pparγ1* allele were mated with ether *ROSA26^CreERT2/CreERT2^* or ROSA26^mTmG/mTmG^ mice. Due to the fact both *Pparγ1* and *Rosa26* localized in the same chromosome, recombinant *Pparγ1^fl^-ROSA26^CreERT2^* and *Pparγ1^fl^-ROSA26^mTmG^* mice were generated. *Pparγ1^fl^-ROSA26^CreERT2^*, *Pparγ1^fl^-ROSA26^mTmG^*, *Pparγ1^wt^-ROSA26^CreERT2^* and *Pparγ1^wt^-ROSA26^mTmG^* mice were then mated with *MMTV-ErbB2* transgenic mice [8,9] to generate *Pparγ1^fl^-ROSA26^CreERT2^MMTV-ErbB2*, *Pparγ1^fl^-ROSA26^mTmG^MMTV-ErbB2*, *Pparγ1^wt^-ROSA26^CreERT2^MMTV-ErbB2* and *Pparγ1^wt^-ROSA26^mTmG^MMTV-ErbB2* mice*. Pparγ1^fl^-ROSA26^CreERT2^Pparγ1^fl^-ROSA26^mTmG^MMTV-ErbB2* (*Pparγ1^fl/fl^CreERT2^+^mTmG^+^MMTV-ErbB2^+^*) and *Pparγ1^wt^-ROSA26^CreERT2^Pparγ1^wt^-ROSA26^mTmG^MMTV-ErbB2* (*Pparγ1^wt/wt^CreERT2^+^mTmG^+^MMTV-ErbB2^+^*) mice were obtained by cross *Pparγ1^fl^-ROSA26^CreERT2^MMTV-ErbB2* with *Pparγ1^fl^-ROSA26^mTmG^MMTV-ErbB2* or *Pparγ1^wt^-ROSA26^CreERT2^MMTV-ErbB2* with *Pparγ1^wt^-ROSA26^mTmG^MMTV-ErbB2* mice. The deletion of *Pparγ1* exon 2 in *Pparγ1^fl/fl^CreERT2^+^mTmG^+^MMTV-ErbB2^+^* mice was induced by intraperitoneal injection of tamoxifen with 75 mg/kg/day for 5 days in 6 weeks-old female mice. The extent of deletion was assessed by PCR-based DNA analysis. *Pparγ1^wt/wt^CreERT2^+^mTmG^+^MMTV-ErbB2^+^* mice were used as control for the potential effect of tamoxifen on mammary gland development and gene expression [94].

### 4.2. Statistics

All means were compared using two-tailed Student’s *t*-test. A *p* value < 0.05 was considered significant. RNA-seq data was aligned using bowtie2 [95] against mm10 genome and RSEM v1.2.12 software [96] was used to estimate gene-level read counts using Ensemble transcriptome information. Raw gene counts were used in DESeq2 [97] algorithm to estimate significance of differential expression between *Pparγ1*^+/+^ and *Pparγ1*^−/−^ groups. Expression heatmaps were plotted using mean-centered log2-scaled normalized by DESeq2 expression values. Gene set enrichment analysis was done using Qiagen’s Ingenuity^®^ Pathway Analysis software (IPA^®^, Qiagen, Redwood City, CA, USA, www.qiagen.com/ingenuity) on genes that passed nominal *p* < 0.05 using “Upstream Regulators” and “Networks” option. Upstream regulators with significantly predicted activation state (|*Z*-score|>1) that in addition passed *p* < 0.05 target enrichment threshold with at least 5 target genes were reported. Identified gene interaction networks with IPA score of at least 25 were considered. Activation states and fold changes were reported for *Pparγ1*^+/+^ condition relative to *Pparγ1*^−/−^. KEGG pathway analysis was conducted as previously described [98]. Analyses of human gene expression derived from The Metastatic Breast Cancer Project (Provisional, February 2020) were conducted using the cBioPortal analytical tools including the Pearson correlation coefficients.

### 4.3. Antibodies

Antibodies used for tumor analysis include F4/80 (ab6640, Abcam, Cambridge, MA, USA) [99], EphA2 (C-20, SC-924, Santa Cruz Biotech, Dallas, TX, USA), Ki-67 (RM-9106-S1, Thermo Fisher Scientific, Waltham, MA, USA), SREBP1 (H-160), SREBP2 (H-164), FASN (H-300), and CD31 (CM303A, Biocare Medical, Concord, CA, USA). Antibodies used for lineage analysis were: anti-B220 (RA3-6B2), anti-CD19 (eBio1D3), anti-IgM µ-chain (Jackson Labs), anti-CD43 (S7) for B cells, anti-CD3 (145-2C11), anti-CD4 (GK1.5), anti-CD8a (53-6.7) for T cells, anti-CD41 (MWReg30), anti-CD71 (C2), anti-Ter119 (TER-119), and anti-F4/80 (BM8), anti-Mac1 (M1/70), and anti-Gr1 (RB6-8C5) for myeloid cells. Antibodies used for HSC and progenitor analysis were: Lineage (biotin-conjugated anti-Gr-1 (RB6-8C5), -Mac1 (M1/70), -B220 (RA3-6B2), -CD19 (eBio1D3), -Ter110 (TER-119), -CD5 (53-7.3), -CD4 (GK1.5), -CD8 (53-6.7)), APC-Cy7-c-Kit (2B8), PerCP-cy5.5-Sca1 (E13-161.7 or D7, 1:1000 dilution), FITC-CD48 (HM48-1), PE-Cy7-CD150 (TC15-12F12.2), APC-CD34 (RAM34) and PE–Flk2 (A2F10.1). All antibodies were used at 1:200 dilution unless otherwise noted. FACS antibodies were purchased from eBioscience (San Diego, CA, USA), BD Biosciences (San Jose, CA, USA) or BioLegend (San Diego, CA, USA).

### 4.4. Cellularity, Hematology and Flow Cytometry

Cellularity of bone marrow, spleen and thymus was calculated from cell count and weights of each organ. Complete blood count (CBC) was measured using a Hemavet 950 (Drew Scientific, Miami Lakes, FL, USA) and hematocrit was calculated after centrifugation of whole blood in heparinized microcapillary tubes (1-000-7500-HC/5 Drummond, Broomall, PA, USA) [57,100]. For flow analysis of lineage distribution in blood, bone marrow, spleen and thymus, red blood cells were first lysed then cells were stained for B, T, and myeloid subsets. All peripheral blood data was acquired using the BD Canto flow cytometer. For bone marrow analysis of HSPC, cells were stained with Lineage (biotin-Ter-119, -Mac-1, -Gr-1, -CD4, -CD8α, -CD5, -CD19 and -B220) followed by staining with streptavidin-PE-TexasRed (SA1017, 1:50, Invitrogen, Carlsbad, CA, USA), and the HSPC panel: -c-Kit-APC, -Sca1-PE, -CD150-PE-Cy7, -CD48-FITC or –c-kit-APC-Cy7, -Sca1-PE-Cy5.5, CD34-APC, and Flk2-PE. Data for bone marrow analysis was collected on the BD Fortessa flow cytometer. Data for bone marrow analysis was collected on the BD Fortessa flow cytometer. All flow cytometry data was analyzed using FlowJo v8.7 for MAC.

### 4.5. Colony Forming Assay

Total BM from femur was plated onto M3434 semi-solid methylcellulose media (STEMCELL Technologies, Vancouver, BC, Canada). Colonies were enumerated 7 days after plating.

### 4.6. Cell Culture, Plasmid DNA, and Transfection

The MCF10A-NeuT cells [101], were cultured in DMEM/Ham’s F-12 (50/50) supplemented with 5% of horse serum, 10 μg/mL of insulin, 20 ng/mL of EGF, 100 ng/mL of Cholera Toxin and 0.5 μg/mL of hydrocortisone. MCF10A-NeuT cells transduced with the PPARγ1 expression vector [78], were maintained in Dulbecco’s Modification of Eagle’s Medium (DMEM) supplemented with 10% fetal bovine serum. A total of 100 μg/mL of each penicillin and streptomycin were included in all media. The mouse PPARγ1 subcloned into p3XFLAG-CMV-10 (Sigma-Aldrich, St. Louis, MO, USA) was previously described [39,78]. The coding region of 3XFLAG-PPARγ1 cDNA was inserted into the retroviral expression vector MSCV-IRES-GFP at the *EcoRI* site (blunted) upstream of the IRES driving expression of GFP [78]. The MCF10A-NeuT cells stably expressing PPARγ1, were established as previously described [102].

### 4.7. Transwell Migration and Xenografts

Transwell migration assay was performed in 24 well transwell inserts (Corning, Tewksbury, MA, USA) as previously described [103,104]. See the supplemental methods for the details. MCF10A-NeuT cells stably expressing PPARγ1, or vector control were previously established described [102] and used in mammary fat pad xenografts as described [105].

### 4.8. Immunohistochemistry (IHC) Staining

IHC staining was performed on the paraffin-embedded tissue blocks in the Kimmel Cancer Center Pathology Core Facility at Thomas Jefferson University. Quantification was conducted using ImageJ software.

### 4.9. ChIP-Seq

For the ChIP-Seq [106], ChIP and input libraries were generated from at least three distinct biological samples from MCF10A-NeuT cells stably expressing vector control or PPARγ1 Wt. Approximately 10 ng of ChIP DNA (quantified by Qubit 2.0 Fluorometer, Invitrogen) was prepared for sequencing according to the amplification protocol from Illumina using enzymes from New England Biolabs (Ipswich, MA, USA) and PCR purification (#28104) and MinElute (#28004) kits from Qiagen. Deep sequencing was performed by the Functional Genomics Core (J. Schugg and K. Kaestner) of the Penn Diabetes Research Center using Illumina HiSeq 2000 and aligned sequences were obtained using the Solexa Analysis Pipeline.

### 4.10. ChIP-Seq Data Analysis

Tag Alignment and Peak Calling. Binding sites for PPAR WT were inferred from ChIP-Seq analysis of chromatin occupancy. ChIP-Seq tags in FASTQ format were mapped to the human genome version hg19 using the bwa-mem aligner with default parameters. ChIP-Seq data analysis was then performed following ENCODE guidelines to generate high quality peak calls [107]. Aligned reads, or “tags,” were filtered based on mapping quality to retain only those with MAPQ score of 30 or higher for further analysis. Within each IP “treatment” group (vector control, PPARγ Wt), peak calling was performed on individual replicates using pooled input as a control using MACS2 peak calling software [108]. Peak calling was also performed on pooled IP “treatment” samples versus pooled input “control” samples within each group. Peak calling was performed using an estimated 150 bp fragment size, and a 10% FDR threshold. MACS2 automatically filters out duplicate reads, and since each individual replicate had less than the recommended 15M unique tags, the MACS2 option “-to-large” was used to linearly scale up read depth to match the number of input tags; the “-to-large” option was not used during the analysis of pooled IP samples. Consistency between ChIP replicates was evaluated using the Irreproducible Discovery Rate (IDR) methodology. Per the IDR framework, individual replicate samples were used to determine the optimal number of peaks in the pooled replicates at an IDR threshold of 5%.

### 4.11. Annotation and Motif Finding

Annotation and motif finding within putative PPARγ binding peaks was performed using the databases and tools provided as part of the Hypergeometric Optimization of Motif EnRichment (HOMER) software suite. The HOMER find Motifs Genome command was used to search peak regions for known and novel motifs. The HOMER annotate Peaks command was used to generate region statistics and identify the closest genomic feature relative to each peak, based on the hg19 genome. The annotate Peaks command was also used in tss-centric, rna-centric, and tts-centric mode to identify the closest peak with respect to transcription start sites, transcribed mRNA regions, and transcription termination sites, respectively.

## 5. Conclusions

Peroxisome proliferator-activated receptor γ (PPARγ) has been implicated in either promoting or inhibiting tumorigenesis, primarily deduced through using agonists of the receptor. Herein, genetic deletion of *Ppar*γ*1* in a murine model of human ErbB2-mediated breast cancer, the rate of tumor progression and death was significantly reduced. Furthermore, the genetic deletion of *Ppar*γ*1* correlated with reduced pro-tumorigenic inflammation and lipogenesis in the mammary tumors of the mice. The induction of local mammary tumor macrophage infiltration occurred without affecting other tissue hematopoietic stem cell pools. Endogenous *Pparγ1* induced expression of both an EphA2-Amphiregulin and an inflammatory INFγ and Cxcl5 signaling module, that was recapitulated in human breast cancer. We conclude ErbB2 collaborates with endogenous Pparγ1 in the onset and progression of mammary tumorigenesis.

## Data Availability

All of the data generated from this study are available upon request.

## Supplementary Materials

The following are available online at https://www.mdpi.com/article/10.3390/cancers13092171/s1, Supplemental materials and methods, Table S1: Test of equality of the survival distribution function (DF = 1), Table S2: Estimated tumor growth rate, Figure S1: Deletion of *Ppar**γ1* reduces adipogenesis in MMTV-ErbB2 transgenic mammary tumors, Figure S2: Growth rate of established ErbB2 mammary tumors upon Cre-mediated deletion of *Ppar**γ1*, Figure S3: Impact of *Ppar**γ1* gene deletion on hematopoietic cell peripheral tissue distribution in multigenic mice, Figure S4: T cell lineage indices of *Pparγ1^+/+^* vs. *Pparγ* ^−/−^ multigenic mice, Figure S5: Colony forming hematopoietic progenitor cell assays.
