# Supplementary material for: Pparγ1 Facilitates ErbB2-Mammary Adenocarcinoma in Mice
Source: Cancers (Basel). 2021 Apr 30;13(9):2171. doi: 10.3390/cancers13092171 (PMC8125290; doi:10.3390/cancers13092171)
Supplement: Supplementary file 1 [file cancers-13-02171-s001.zip › cancers-1168948 - supplementary materials.pdf]

cancers

MDPI

Supplementary Files

Pparγ1 Facilitates ErbB2-Mammary Adenocarcinoma in Mice

Xuanmao Jiao, Lifeng Tian, Zhao Zhang, Joanna Balcerek, Andrew V. Kossenkov, Mathew C. Casimiro, Chenguang Wang, Yichuan Liu, Adam Ertel, Raymond E. Soccio, Eric Chen, Qin Liu, Anthony W. Ashton, Wei Tong and Richard G. Pestell

1. Supplemental Materials and Methods

1.1. Transgenic Mice

The appropriate Thomas Jefferson University institutional committee approved protocols were followed when working with all mice. Animals were housed and maintained at Thomas Jefferson University in a pathogen-free barrier facility under National Institutes of Health (NIH) guidelines. Mice were kept on a 12 hours light/dark cycle with *ad libitum* access to chow and water. The *Pparγ1*$^{fl/fl}$ mice (which remove exon 2 of the *Pparγ1* locus [1]), ROSA26^{CreERT2} mice (Expressing CRE-ERT2 fusion protein under the control of the ubiquitous ROSA26 promoter. Cre-ERT2 fusion protein will bind with Tamoxifen and then transport to nucleus to induce the deletion of floxed alleles [2]) and ROSA26^{mTmG} mice [3] (ROSA26^{mTmG} is a cell membrane-targeted, two-color fluorescent Cre-reporter allele. Prior to Cre recombination, tdTomato (mT) red fluorescence will be expressed. Followed Cre recombination, instead of the red fluorescence, cell membrane-localized EGFP (mG) fluorescence will be expressed) (The Jackson Laboratory, Stock No. 007576) [3] were previously described. Mice homozygous for a floxed *Pparγ1* allele were mated with ether *ROSA26*$^{CreERT2/CreERT2}$ or ROSA26$^{mTmG/mTmG}$ mice. Due to both Pparγ1 and Rosa26 localized in the same chromosome, recombinant *Pparγ1*^{fl}*-ROSA26*^{CreERT2} and *Pparγ1*^{fl}*-ROSA26*^{mTmG} mice were generated. *Pparγ1*^{fl}*-ROSA26*^{CreERT2}, *Pparγ1*^{fl}*-ROSA26*^{mTmG}, *Pparγ1*^{wt}*-ROSA26*^{CreERT2} and *Pparγ1*^{wt}*-ROSA26*^{mTmG} mice were then mated with *MMTV-ErbB2* transgenic mice [4,5] to generate *Pparγ1*^{fl}*-ROSA26*^{CreERT2}*MMTV-ErbB2*, *Pparγ1*^{fl}*-ROSA26*^{mTmG}*MMTV-ErbB2*, *Pparγ1*^{wt}*-ROSA26*^{CreERT2}*MMTV-ErbB2* and *Pparγ1*^{wt}*-ROSA26*^{mTmG}*MMTV-ErbB2 mice. Pparγ1*^{fl}*-ROSA26* ^{CreERT2}*Pparγ1*^{fl}*-ROSA26*^{mTmG}*MMTV-ErbB2* (*Pparg1*$^{fl/fl}$*Cre-ERT2*$^{+}$*mTmG*$^{+}$*MMTV-ErbB2*$^{+}$) *and Pparγ1*^{wt}*-ROSA26*^{CreERT2}*Pparγ1*^{wt}*-ROSA26*^{mTmG}*MMTV-ErbB2* (*Pparg1*$^{wt/wt}$*CreERT2*$^{+}$*mTmG*$^{+}$*MMTV-ErbB2*$^{+}$) mice were got by cross *Pparγ1*^{fl}*-ROSA26*^{CreERT2}*MMTV-ErbB2* with *Pparγ1*^{fl}*-ROSA26*^{mTmG}*MMTV-ErbB2* or *Pparγ1*^{wt}*-ROSA26*^{CreERT2}*MMTV-ErbB2* with *Pparγ1*^{wt}*-ROSA26*^{mTmG}*MMTV-ErbB2 mice.* The deletion of *Pparγ1* exon 2 in *Pparg1*$^{fl/fl}$*CreERT2*$^{+}$*mTmG*$^{+}$*MMTV-ErbB2*$^{+}$ mice was induced by intraperitoneal injection of Tamoxifen with 75 mg/kg/day for 5 days in 6 weeks-old female mice. The extent of deletion was assessed by PCR-based DNA analysis. *Pparg1*$^{wt/wt}$*Cre-ERT2*$^{+}$*mTmG*$^{+}$*MMTV-ErbB2*$^{+}$ mice were used as control for the potential effect of tamoxifen on mammary gland development and gene expression [6]. Mammary squashes and analysis of mammary gland development were conducted as previously described [7].

1.2. Statistics

All means were compared using two-tailed student's t-test. A p value < 0.05 was considered significant. RNA-seq data was aligned using bowtie2 [8] against mm10 genome and RSEM v1.2.12 software [9] was used to estimate gene-level read counts using Ensemble transcriptome information. Raw gene counts were used in DESeq2 [10] algorithm to estimate significance of differential expression *Pparγ1*$^{+/+}$ and *Pparγ1*$^{-/-}$ groups. Expression heatmaps were plotted using mean-centered log2-scaled normalized by DESeq2 expression values. Putative regulator gene set enrichment analysis was done using QIAGEN's Ingenuity® Pathway Analysis software (IPA®, QIAGEN Redwood City, CA, USA www.qiagen.com/ingenuity) on genes that passed nominal $p < 0.05$ using "Upstream Regulators" option. Upstream regulators with significantly predicted activation state (|Z-

score| > 1) that in addition passed $p < 0.05$ target enrichment threshold with at least 5 target genes were reported. Activation state and fold changes were reported for *Pparγ1*$^{+/+}$ condition relative to *Pparγ1*$^{-/-}$.

1.3. Antibodies

Antibodies used for lineage analysis were: SREBP1 (H-160), SREBP2 (H-164), FASN (H-300), PPARγ (H-100), PPARγ (E-8), anti-B220 (RA3-6B2), anti-CD19 (eBio1D3), anti-IgM μ-chain (Jackson labs), anti-CD43 (S7) for B cells, anti-CD3 (145-2C11), anti-CD4 (GK1.5), anti-CD8a (53-6.7) for T cells, anti-CD41 (MWReg30), anti- CD71 (C2), anti-Ter119 (TER-119), and anti-F4/80 (BM8), anti-Mac1 (M1/70), and anti-Gr1 (RB6-8C5) for myeloid cells. Antibodies used for HSC and progenitor analysis were: Lineage (biotin-conjugated anti-Gr-1 (RB6-8C5), -Mac1 (M1/70), -B220 (RA3-6B2), -CD19 (eBio1D3), -Ter110 (TER-119), -CD5 (53-7.3), -CD4 (GK1.5), -CD8 (53-6.7), APC-Cy7-c-Kit (2B8), PerCP-cy5.5-Sca1 (E13-161.7 or D7, 1:1000 dilution), FITC -CD48 (HM48-1), PE-Cy7-CD150 (TC15-12F12.2), APC -CD34 (RAM34) and PE–Flk2 (A2F10.1). All antibodies were used at 1:200 dilution unless otherwise noted. FACS antibodies were purchased from eBioscience (San Diego, CA, USA), BD Biosciences (San Jose, CA, USA) or BioLegend (San Diego, CA, USA).

1.4. Cellularity, Hematology and Flow Cytometry

Cellularity of bone marrow, spleen and thymus was calculated from cell count and weights of each organ. Complete blood count (CBC) was measured using a Hemavet 950 (Drew Scientific, Miami Lakes, FL, USA) and hematocrit was calculated after centrifugation of whole blood in heparinized microcapillary tubes (1-000-7500-HC/5 Drummond, Broomall, PA, USA) [11,12]. For flow analysis of lineage distribution in blood, bone marrow, spleen and thymus, red blood cells were first lysed then cells were stained for B, T, and myeloid subsets. All peripheral blood data was acquired using the BD Canto flow cytometer. For bone marrow analysis of HSPC, cells were stained with Lineage (biotin-Ter-119, -Mac-1, -Gr-1, -CD4, -CD8α, -CD5, -CD19 and -B220) followed by staining with streptavidin-PE-TexasRed (SA1017, 1:50, Invitrogen, Carlsbad, CA, USA), and the HSPC panel: -c-Kit-APC, -Sca1-PE, -CD150-PE-Cy7, -CD48-FITC or –c-kit-APC-Cy7, -Sca1-PE-Cy5.5, CD34-APC, and Flk2-PE. Data for bone marrow analysis was collected on the BD Fortessa flow cytometer. Data for bone marrow analysis was collected on the BD Fortessa flow cytometer. All flow cytometry data was analyzed using FlowJo v8.7 for MAC.

1.5. Cell Culture, Plasmid DNA, and Transfection

MCF10A-NeuT [13], were cultured in DMEM: Ham's F-12 (50 of 50) supplemented with 5% of horse serum, 10 μg/mL of insulin, 20 ng/mL of EGF, 100 ng/mL of Cholera Toxin and 0.5 μg/mL of hydrocortisone. MCF10A-NeuT cells transduced with PPARγ [14], were maintained in Dulbecco's Modification of Eagle's Medium (DMEM) supplemented with 10% fetal bovine serum. A total of 100 μg/mL of each penicillin and streptomycin were included in all media. All cells were cultured in humidified atmosphere with 5% CO_2 at 37 °C. The mouse PPARγ subcloned into p3XFLAG-CMV-10 (Sigma-Aldrich, St. Louis, MO, USA) was previously described [14,15]. The integrity of all constructs was confirmed by sequence analysis. The MCF10A-NeuT cells stably expressing PPARγ Wt was established as previously described [16].

1.6. Colony Forming Assay

Total BM from femur was plated onto M3434 semi-solid methylcellulose media (STEMCELL Technologies, Vancouver, BC, Canada). Colonies were enumerated 10–14 days after plating.

1.7. Tranwell Migration Assay

Transwell migration assays were performed as described before [17,18]. In brief, transwell inserts (8 mM pore size) were coated with Matrigel (50 mg/mL) overnight at 37 °C in a humidified incubator. The following day cells were non-enzymatically harvested by incubation with cell stripper solution (Corning) at 37 °C. Cells were centrifuged at 100 × *g* for 5 min and cell pellets suspended at 1×10^{6} cells/mL in 0.1% (v/v) FBS in DMEM. Matrigel solution was removed from transwell inserts, cells (1×10^5/insert) added to the upper chamber and incubated for 1 h to attach to transwell inserts. To initiate migration inserts were moved into wells containing 10% (v/v) FBS in DMEM and incubated at 37 °C for 5 h. At the conclusion of the experiment non-migrated cells were removed from the upper surface of the transwell using a cotton bud. Membranes were fixed in 1% (w/v) formaldehyde in PBS for 10 min and stained using 0.5% (w/v) methylene blue according to previous reports [18]. Cells were imaged using an inverted microscope (Nikon Corp) and 8 Mp digital camera.

1.8. Western Blot Analysis

Whole cell lysates (50 µg) from cell culture or tumor samples were separated by SDS-PAGE and the proteins transferred to nitrocellulose membrane for Western blotting as previously described [15]. Guanine nucleotide dissociation inhibitor (GDI), or Vinculin was used as internal protein loading control. Densitometry was performed using Alpha-Imager software. In brief, the intensity of specific band was subtracted with blank area, and then was normalized to loading control.

1.9. Immunohistochemistry (IHC) Staining

IHC staining was performed on the paraffin-embedded tissue blocks in the Kimmel Cancer Center Pathology Core Facility at Thomas Jefferson University. Quantification was conducted using ImageJ software.

1.10. ChIP and ChIP-Seq

ChIP and CHIP-seq were described previously [19]. Briefly, cell lysis and chromatin fragmentation on cross-linked whole-cells was prepared in ChIP buffer (50 mM Tris-HCL pH 7.5, 140 mM NaCl, 1 mM EDTA, 1% Triton X-100, 0.1% NaDOC, 0.1% SDS) with protease inhibitors (1 mM PMSF and Complete protease inhibitor tablet from Roche) by probe sonication (F60 Sonic Dismembrator, Fisher Scientific, Waltham, MA, USA). Samples were sonicated at low power (level 4) for three pulses of 10 seconds each and at a higher power (level 6) for two pulses of 10 seconds each. Proteins were bound at 4°C overnight with 10 µg anti-PPARγ (sc-7196, Santa Cruz Biotechnologies) in ChIP buffer with protease inhibitors and then immunoprecipitated with Protein A Sepharose Beads (GE Healthcare). Beads were washed for 5 minutes each with the following buffers: ChIP Buffer, mixed Micelle buffer (150 mM NaCl, 20 mM Tris-HCl pH 8.0, 5 mM EDTA, 5.2% Sucrose, 1% Triton X-100, 0.2% SDS), Buffer 500 (250 mM NaCl, 5 mM Tris-HCl pH 8.0, 0.5 mM EDTA, 25 mM HEPES, 0.5% Triton X-100, 0.05% NaDOC), LiCl/Detergent Buffer (250 mM LiCl, 10 mM Tris-HCl pH 8.0, 10 mM EDTA, 0.5% IGEPAL CA-630, 0.5% NaDOC), and Tris-EDTA pH 8.0. Cross-linking was reversed at 65°C overnight in elution buffer (50 mM Tris-HCl pH 8.0, 10 mM EDTA, 1% SDS) and DNA was isolated using phenol/chloroform extraction and NaCl/EtOH precipitation with 20 µg glycogen carriers. DNA was subsequently analyzed by quantitative PCR using ABI 7500 Fast Real-Time PCR System and Power SYBR Green PCR Master Mix (Applied Biosystems).

For the ChIP-Seq, ChIP and input libraries were generated from at least three distinct biological samples from MCF10A-NeuT cells stably expressing vector control, or PPARγ Wt. Approximately 10 ng of ChIP DNA (quantified by Qubit 2.0 Fluorometer, Invitrogen) was prepared for sequencing according to the amplification protocol from Illumina using enzymes from New England Biolabs (Ipswich, MA, USA)and PCR purification (#28104)

and MinElute (#28004) kits from Qiagen. Deep sequencing was performed by the Functional Genomics Core (J. Schugg and K. Kaestner) of the Penn Diabetes Research Center using Illumina HiSeq 2000 and aligned sequences were obtained using the Solexa Analysis Pipeline.

1.11. ChIP-Seq Data Analysis

Tag Alignment and Peak Calling. Binding sites for PPARγ WT were inferred from ChIP-Seq analysis of chromatin occupancy. ChIP-Seq tags in FASTQ format were mapped to the human genome version hg19 using the bwa-mem aligner with default parameters. ChIP-Seq data analysis was then performed following ENCODE guidelines to generate high quality peak calls (20). Aligned reads, or "tags," were filtered based on mapping quality to retain only those with MAPQ score of 30 or higher for further analysis. Within each IP "treatment" group (Vector control, PPARγ Wt), peak calling was performed on individual replicates using pooled input as a control using MACS2 peak calling software (21). Peak calling was also performed on pooled IP "treatment" samples versus pooled input "control" samples within each group. Peak calling was performed using an estimated 150 bp fragment size, and a 10% FDR threshold. MACS2 automatically filters out duplicate reads, and since each individual replicate had less than the recommended 15M unique tags, the MACS2 option "-to-large" was used to linearly scale up read depth to match the number of input tags; the "-to-large" option was not used during the analysis of pooled IP samples. Consistency between ChIP replicates was evaluated using the Irreproducible Discovery Rate (IDR) methodology. Per the IDR framework, individual replicate samples were used to determine the optimal number of peaks in the pooled replicates at an IDR threshold of 5%.

2. Supplementary Files

Table S1. Test of equality of the survival distribution function (DF = 1).

Statistic	Observed	Critical Value	*p*-Value	Alpha
Log-rank	4.099	3.841	0.043	0.050
Wilcoxon	4.829	3.841	0.028	0.050
Tarone-Ware	4.530	3.841	0.033	0.050

Table S2. Estimated tumor growth rate. Mixed effects models were used to estimate the tumor growth rate for each group. Tumor volume was log2-transformed for data analysis. Estimated tumor growth rate reflected using days needed for specific fold changes.

Group	Days for 2-Fold Change	Days for 4-Fold Change	Days for 16-Fold Change
WT	4.6	9.2	18.4
KO	5.8	11.7	23.4

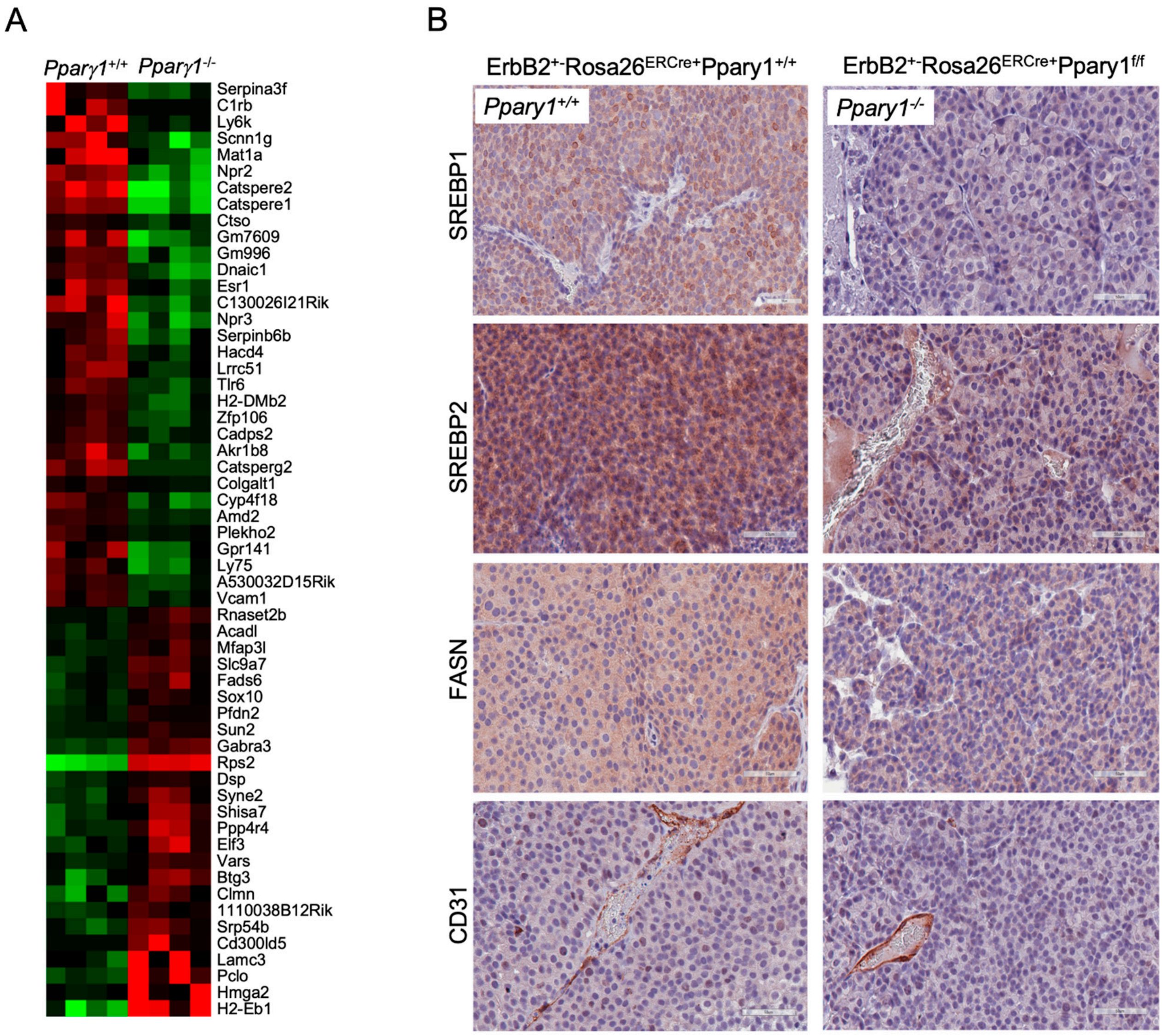

Figure S1. Deletion of *Pparγ1* reduces adipogenesis in MMTV-ErbB2 transgenic mammary tumors. (**A**) Gene expression profile from tumors of ErbB2 mammary OncoMice with inducible *Pparγ1* deletion. (**B**) Representative staining of tumors derived from the transgenic mice for SREBP1, SREBP2, FASN, and CD31 shown for each genotype.

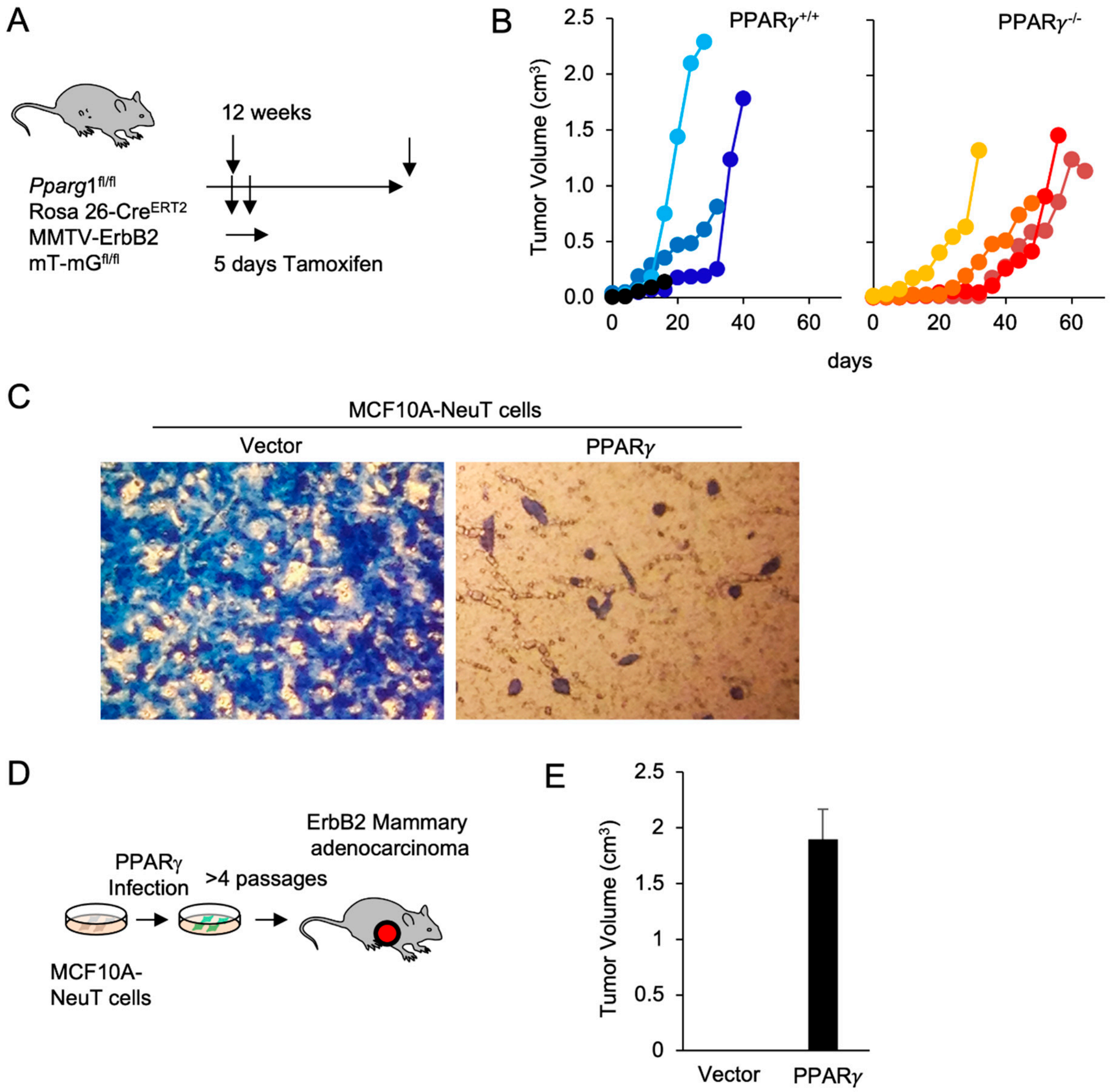

Figure S2. Growth rate of established ErbB2 mammary tumors upon Cre-mediated deletion of *Pparγ1*. (**A**) Schematic representation of the transgenic mouse genotype and the timing of tamoxifen treatment to induce Cre-mediated *Pparg1* gene excision in mice with early established mammary tumors at 12 weeks. (**B**) Tumor volume in MMTV-ErbB2, $ROSA26^{CreERT2/mTmG}$ mice that were either $Ppar\gamma 1^{+/+}$ or $Ppar\gamma 1^{-/-}$ (KO) for $n = 4$ of each genotype. (**C**) Transwell migration assays comparing MCF10A-NeuT-Pparg1 vs. MCF10A-NeuT-vector. (**D**) Schematic representation of the xenograft tumor model of MCF10A-NeuT cells. MCF10A-NeuT cells were transduced with PPARγ Wt or vector control as indicated. 5×10^6 cells were implanted into the mammary fat pad of immune-deficient nude mice. (E)Tumor growth was measured at 20 days by digital caliper, and tumor volume was calculated. Data are shown as mean ± SEM for $n = 13$.

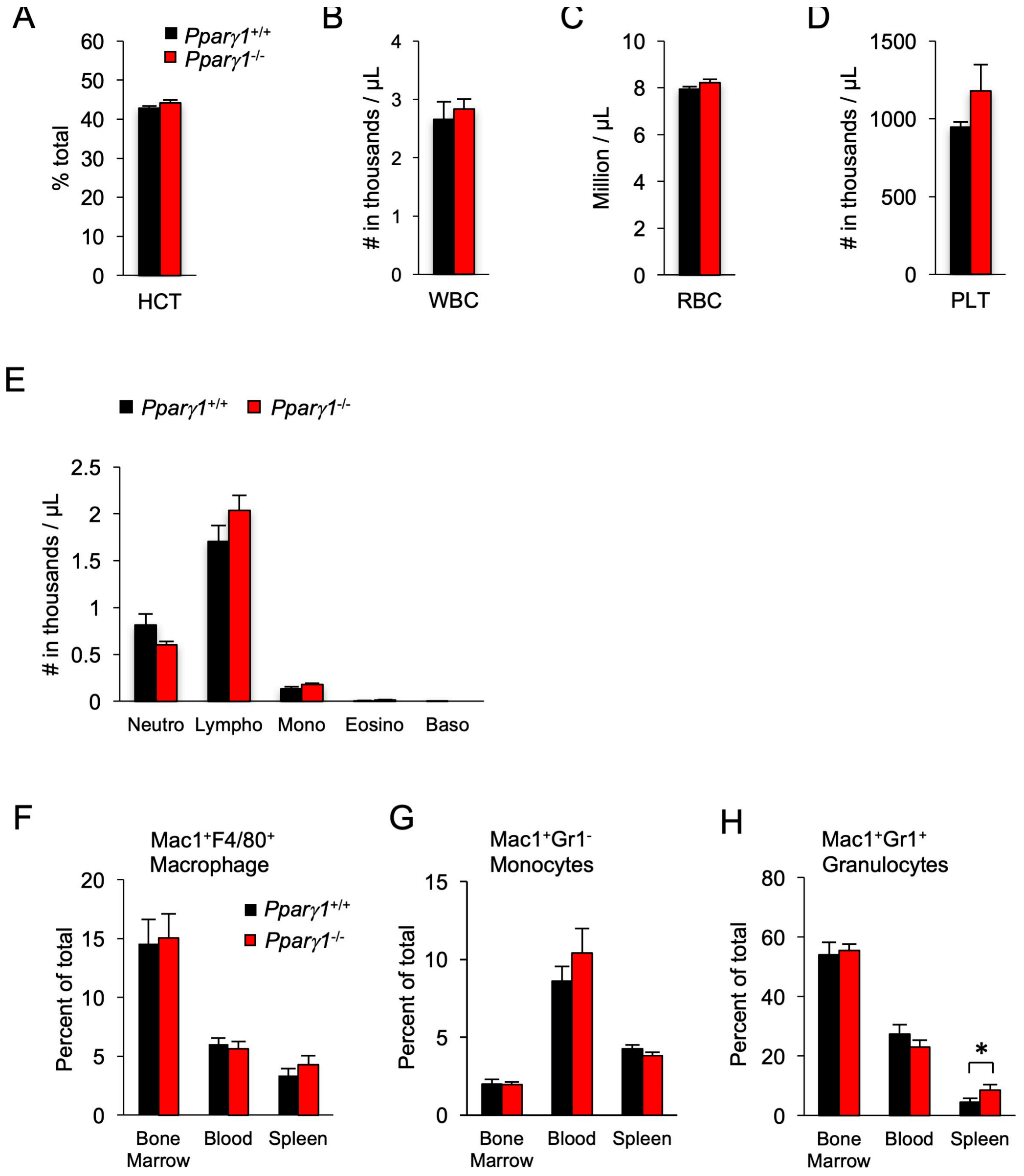

Figure S3. Impact of *Pparγ1* gene deletion on hematopoietic cell peripheral tissue distribution in multigenic mice (*Pparγ1*$^{wt/wt}$, vs. *Pparγ1*$^{fl/fl}$, both in *MMTV-ErbB2, ROSA26*$^{CreERT2/mTmG}$ background and treated with tamoxifen). (**A**–**D**). Peripheral blood indices for hematocrit (HCT) (**A**), white blood cells (WBC) (**B**), red blood cells (RBC) (**C**) and platelets (PLT) (**D**,**E**). The relative number of WBC subtypes with data shown as mean ± SEM for $n = 5$ each group. (**F**–**H**). Relative distribution of macrophages (**F**), monocytes (**G**), and granulocytes (**H**) in bone marrow, blood and spleen. Data are shown as mean ± SEM and p values are calculated by two-tailed Students' t-test.

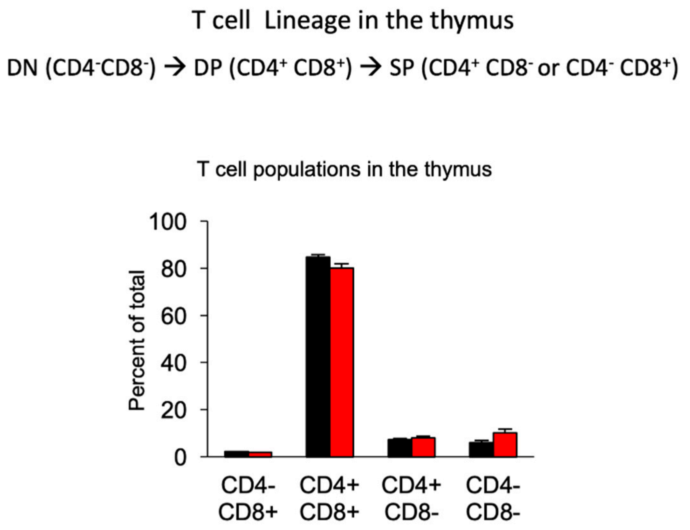

Figure S4. T cell lineage indices of *Pparγ1*$^{+/+}$ *vs*. *Pparγ1*$^{-/-}$ multigenic mice (*Pparγ1*$^{wt/wt}$ vs. *Pparγ1*$^{fl/fl}$, both in *MMTV-ErbB2, ROSA26*^{CreERT2}/^{mTmG} background and treated with tamoxifen). Relative proportion of CD4/CD8 T lineage components in the thymus. Data are shown as mean ± SEM and *p* values are calculated by two-tailed Students' *t*-test.

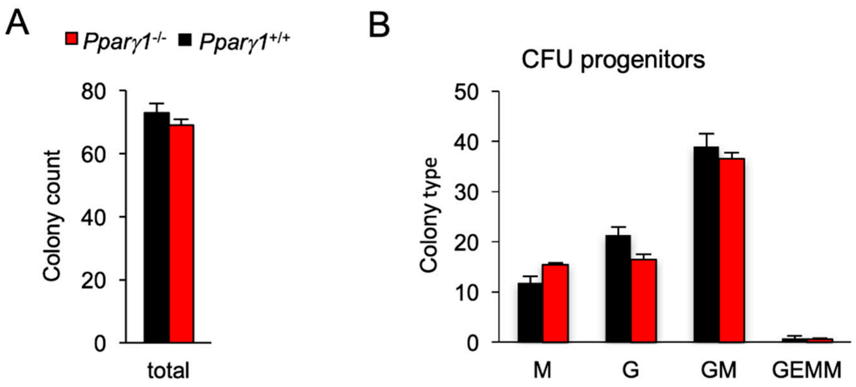

Figure S5. Colony forming hematopoietic progenitor cell assays. (**A**) Colonies numbers formed from *Pparγ1*$^{+/+}$ vs. *Pparγ1*$^{-/-}$ shown as mean ± SEM for *n* = 5 with (**B**). colony type shown as mean ± SEM in graphical form.

References

1. Akiyama, T.E.; Sakai, S.; Lambert, G.; Nicol, C.J.; Matsusue, K.; Pimprale, S.; Lee, Y.-H.; Ricote, M.; Glass, C.K.; Brewer, H.B.; et al. Conditional Disruption of the Peroxisome Proliferator-Activated Receptor γ Gene in Mice Results in Lowered Expression of ABCA1, ABCG1, and apoE in Macrophages and Reduced Cholesterol Efflux. *Mol. Cell. Biol.* **2002**, *22*, 2607–2619, doi:10.1128/mcb.22.8.2607-2619.2002.
2. De Luca, C.; Kowalski, T.J.; Zhang, Y.; Elmquist, J.K.; Lee, C.; Kilimann, M.W.; Ludwig, T.; Liu, S.-M.; Chua, S.C., Jr. Complete rescue of obesity, diabetes, and infertility in db/db mice by neuron-specific LEPR-B transgenes. *J. Clin. Investig.* **2005**, *115*, 3484–3493, doi:10.1172/jci24059.
3. Muzumdar, M.D.; Tasic, B.; Miyamichi, K.; Li, L.; Luo, L. A global double-fluorescent Cre reporter mouse. *Genes* **2007**, *45*, 593–605, doi:10.1002/dvg.20335.
4. Hulit, J.; Lee, R.J.; Li, Z.; Wang, C.; Katiyar, S.; Yang, J.; Quong, A.A.; Wu, K.; Albanese, C.; Russell, R.; et al. p27Kip1 repression of ErbB2-induced mammary tumor growth in transgenic mice involves Skp2 and Wnt/beta-catenin signaling. *Cancer Res.* **2006**, *66*, 8529–8541.
5. Lee, R.J.; Albanese, C.; Fu, M.; D'Amico, M.; Lin, B.; Watanabe, G.; Haines, G.K.; Siegel, P.M.; Hung, M.-C.; Yarden, Y.; et al. Cyclin D1 Is Required for Transformation by Activated Neu and Is Induced through an E2F-Dependent Signaling Pathway. *Mol. Cell. Biol.* **2000**, *20*, 672–683, doi:10.1128/mcb.20.2.672-683.2000.

6. Shehata, M.; Van Amerongen, R.; Zeeman, A.L.; Giraddi, R.R.; Stingl, J. The influence of tamoxifen on normal mouse mammary gland homeostasis. *Breast Cancer Res.* **2014**, *16*, 1–11, doi:10.1186/s13058-014-0411-0.
7. Rowlands, T. M., Pechenkina, I. V., Hatsell, S. J., Pestell, R. G., & Cowin, P.. Dissecting the roles of beta-catenin and cyclin D1 during mammary development and neoplasia. Proceedings of the National Academy of Sciences of the United States of America, **2003**, *100(20)*, 11400–11405, doi:10.1073/pnas.1534601100
8. Langmead, B.; Salzberg, S.L. Fast gapped-read alignment with Bowtie 2. *Nat. Methods* **2012**, *9*, 357–359, doi:10.1038/nmeth.1923.
9. Li, B.; Dewey, C.N. RSEM: Accurate transcript quantification from RNA-Seq data with or without a reference genome. *BMC Bioinform.* **2011**, *12*, 323, doi:10.1186/1471-2105-12-323.
10. Love, M.I.; Huber, W.; Anders, S. Moderated estimation of fold change and dispersion for RNA-seq data with DESeq2. *Genome Biol.* **2014**, *15*, 550.
11. Balcerek, J.; Jiang, J.; Li, Y.; Jiang, Q.; Holdreith, N.; Singh, B.; Chandra, V.; Lv, K.; Ren, J.-G.; Rozenova, K.; et al. Lnk/Sh2b3 deficiency restores hematopoietic stem cell function and genome integrity in Fancd2 deficient Fanconi anemia. *Nat. Commun.* **2018**, *9*, 1–14, doi:10.1038/s41467-018-06380-1.
12. Lv, K.; Jiang, J.; Donaghy, R.; Riling, C.R.; Cheng, Y.; Chandra, V.; Rozenova, K.; An, W.; Mohapatra, B.C.; Goetz, B.T.; et al. CBL family E3 ubiquitin ligases control JAK2 ubiquitination and stability in hematopoietic stem cells and myeloid malignancies. *Genes Dev.* **2017**, *31*, 1007–1023, doi:10.1101/gad.297135.117.
13. Liu, M.; Casimiro, M.C.; Wang, C.; Shirley, L.A.; Jiao, X.; Katiyar, S.; Ju, X.; Li, Z.; Yu, Z.; Zhou, J.; et al. p21CIP1attenuates Ras- and c-Myc-dependent breast tumor epithelial mesenchymal transition and cancer stem cell-like gene expression in vivo. *Proc. Natl. Acad. Sci. USA* **2009**, *106*, 19035–19039, doi:10.1073/pnas.0910009106.
14. Tian, L.; Zhou, J.; Casimiro, M.C.; Liang, B.; Ojeifo, J.O.; Wang, M.; Hyslop, T.; Wang, C.; Pestell, R.G. Activating Peroxisome Proliferator-Activated Receptor γ Mutant Promotes Tumor Growth In vivo by Enhancing Angiogenesis. *Cancer Res.* **2009**, *69*, 9236–9244, doi:10.1158/0008-5472.can-09-2067.
15. Zhou, J.; Zhang, W.; Liang, B.; Casimiro, M.C.; Whitaker-Menezes, D.; Wang, M.; Lisanti, M.P.; Lanza-Jacoby, S.; Pestell, R.G.; Wang, C. PPARγ activation induces autophagy in breast cancer cells. *Int. J. Biochem. Cell Biol.* **2009**, *41*, 2334–2342, doi:10.1016/j.biocel.2009.06.007.
16. Tian, L.; Wang, C.; Hagen, F.K.; Gormley, M.; Addya, S.; Soccio, R.; Casimiro, M.C.; Zhou, J.; Powell, M.J.; Xu, P.; et al. Acetylation-defective mutants of Pparγ are associated with decreased lipid synthesis in breast cancer cells. *Oncotarget* **2014**, *5*, 7303–7315, doi:10.18632/oncotarget.2371.
17. Li, Z.; Jiao, X.; Wang, C.; Ju, X.; Lu, Y.; Yuan, L.; Lisanti, M.P.; Katiyar, S.; Pestell, R.G. Cyclin D1 Induction of Cellular Migration Requires p27KIP1. *Cancer Res.* **2006**, *66*, 9986–9994, doi:10.1158/0008-5472.can-06-1596.
18. Jiao, X.; Wang, M.; Zhang, Z.; Li, Z.; Ni, D.; Ashton, A.W.; Tang, H.-Y.; Speicher, D.W.; Pestell, R.G. Leronlimab, a humanized monoclonal antibody to CCR5, blocks breast cancer cellular metastasis and enhances cell death induced by DNA damaging chemotherapy. *Breast Cancer Res.* **2021**, *23*, 1–15, doi:10.1186/s13058-021-01391-1.
19. Casimiro, M.C.; Crosariol, M.; Loro, E.; Ertel, A.; Yu, Z.; Dampier, W.; Saria, E.A.; Papanikolaou, A.; Stanek, T.J.; Li, Z.; et al. ChIP sequencing of cyclin D1 reveals a transcriptional role in chromosomal instability in mice. *J. Clin. Investig.* **2012**, *122*, 833–843, doi:10.1172/jci60256.
20. Landt, S.G.; Marinov, G.K.; Kundaje, A.; Kheradpour, P.; Pauli, F.; Batzoglou, S.; Bernstein, B.E.; Bickel, P.; Brown, J.B.; Cayting, P.; et al. ChIP-seq guidelines and practices of the ENCODE and modENCODE consortia. *Genome Res.* **2012**, *22*, 1813–1831.
21. Zhang, Y.; Liu, T.; Meyer, C.A.; Eeckhoute, J.; Johnson, D.S.; Bernstein, B.E.; Nusbaum, C.; Myers, R.M.; Brown, M.; Lei, W.; et al. Model-based analysis of ChIP-Seq (MACS). *Genome Biol.* **2008**, *9*, R137.
